# Optogenetic control of apical constriction induces synthetic morphogenesis in mammalian tissues

**DOI:** 10.1038/s41467-022-33115-0

**Published:** 2022-09-14

**Authors:** Guillermo Martínez-Ara, Núria Taberner, Mami Takayama, Elissavet Sandaltzopoulou, Casandra E. Villava, Miquel Bosch-Padrós, Nozomu Takata, Xavier Trepat, Mototsugu Eiraku, Miki Ebisuya

**Affiliations:** 1 European Molecular Biology Laboratory (EMBL) Barcelona, Dr. Aiguader 88, 08003 Barcelona, Spain; 2 RIKEN Center for Biosystems Dynamics Research (RIKEN BDR), 2-2-3 Minatojima-minamimachi, Chuo-ku, 650-0047 Kobe, Japan; 3 Institute for Bioengineering of Catalonia (IBEC), The Barcelona Institute for Science and Technology (BIST), Barcelona, Spain; 4 Centro de Investigación Biomédica en Red en Bioingeniería, Biomateriales y Nanomedicina (CIBER-BBN), Barcelona, Spain; 5 Institució Catalana de Recerca i Estudis Avançats (ICREA), Barcelona, Spain

**Keywords:** Optogenetics, Cytoskeleton, Morphogenesis

## Abstract

The emerging field of synthetic developmental biology proposes bottom-up approaches to examine the contribution of each cellular process to complex morphogenesis. However, the shortage of tools to manipulate three-dimensional (3D) shapes of mammalian tissues hinders the progress of the field. Here we report the development of OptoShroom3, an optogenetic tool that achieves fast spatiotemporal control of apical constriction in mammalian epithelia. Activation of OptoShroom3 through illumination in an epithelial Madin-Darby Canine Kidney (MDCK) cell sheet reduces the apical surface of the stimulated cells and causes displacements in the adjacent regions. Light-induced apical constriction provokes the folding of epithelial cell colonies on soft gels. Its application to murine and human neural organoids leads to thickening of neuroepithelia, apical lumen reduction in optic vesicles, and flattening in neuroectodermal tissues. These results show that spatiotemporal control of apical constriction can trigger several types of 3D deformation depending on the initial tissue context.

## Abstract (web summary)

Apical constriction is a force-generating process required during embryonic development; there is a lack of tools to manipulate 3D shapes of mammalian tissues. Here the authors report the optogenetic method, OptoShroom3, to achieve fast spatiotemporal control of apical constriction in mammalian epithelia.

## Introduction

Morphogenesis is the process by which cells organize to form 3D tissues and organs. The study of developing embryos has identified cell-level mechanisms that need to be coordinated to achieve morphogenesis. However, it is difficult to test the sufficiency of a mechanism to cause a specific change in tissue structure and to study feedback between multiple mechanisms during complex embryogenesis. As a solution, the field of synthetic morphology^1^ or synthetic developmental biology^2–6^ proposes to reconstitute morphogenetic events in vitro by gaining control of the constituent cell-level mechanisms.

Apical constriction, a process by which a cell actively reduces its apical surface, is necessary for the formation of numerous curved structures in metazoan embryos^7,8^. The driving force of apical constriction is actomyosin contraction, which is often triggered by activation of the Rho-ROCK pathway on the apical side. Because apical constriction occurs in specific stages and areas of the developing embryo, reconstituting curved tissues requires tools to control cellular contractility in space and time. Optogenetics is a powerful methodology to gain spatiotemporal control of biological processes from the molecular to the multicellular level^9–17^. Izquierdo et al. employed optogenetics to recruit RhoGEF to the plasma membrane in *Drosophila* embryos. Selective optogenetic activation on the apical side of dorsal cells led to tissue invagination, demonstrating that apical constriction is sufficient to induce deformation in that context^18^. Similar tools have been developed to spatiotemporally increase or reduce contractility in mammalian cells, mainly through recruiting RhoGEF, RhoA, or myosin regulators to the plasma membrane. The approach has been effectively used to study mechanotransduction^19^, cell junction remodeling^20^, cytoskeletal dynamics^21^, and cytokinesis^22^. However, this approach has been mainly applied to study cell-level events, and its application to induce morphogenesis in complex tissue shapes is technically challenging. Since all previous tools have been based on recruitment to the plasma membrane, they require a precise multi-photon stimulation of the apical membrane to induce constriction of the apical side only. Therefore, there is still a lack of tools to manipulate 3D tissue deformation and reconstitute mammalian morphogenesis.

In addition, the recent development of organoids, stem cell-derived 3D structures^23–25^, offers unique opportunities to study the interplay between tissue shape and function in vitro. However, the manipulation of organoid shape with optogenetic tools remains unexplored.

In this study, we present an optogenetic tool that achieves control of apical constriction in mammalian cells, inducing multiple types of 3D tissue deformation. The tool is based on Shroom3, a key regulator of apical constriction necessary for several morphogenetic processes in vertebrates, including neural tube closure, lens placode invagination, and morphogenesis of gut and kidney^26–30^. The optogenetic version of Shroom3, OptoShroom3, is capable of fast activation and deactivation of apical constriction at the cell level. We demonstrate that an increase in apical tension causes tissue folding, thickening, flattening, and lumen shrinkage in epithelial cell sheets and neural organoids.

## Results

### Development of OptoShroom3 to control apical constriction

**Fig. 1:**
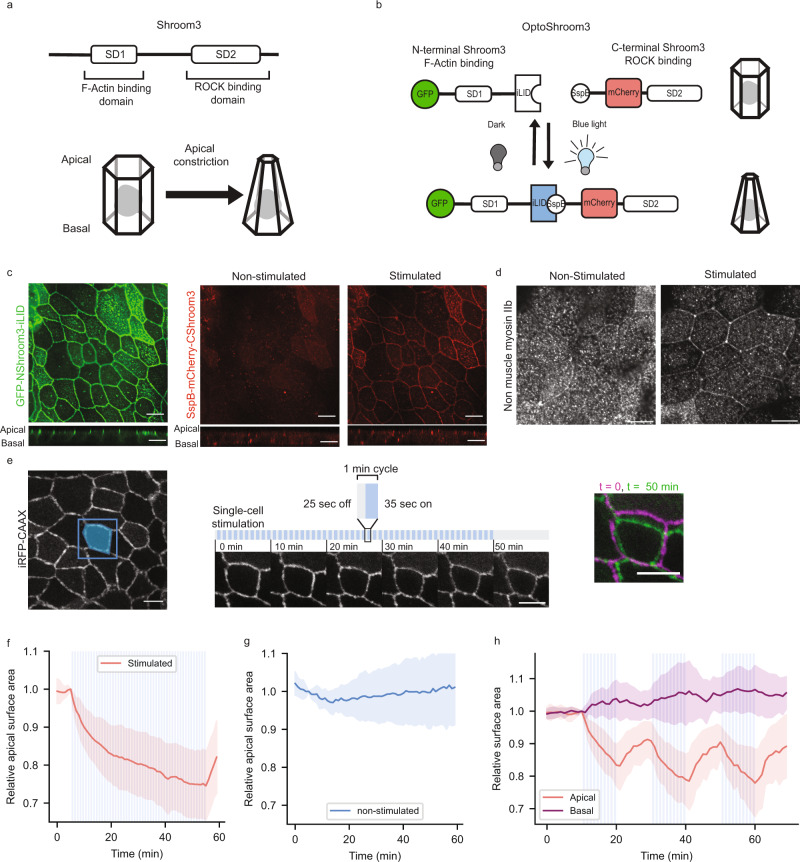
Characterization of OptoShroom3-induced apical constriction. **a** Protein structure of wild-type Shroom3 and scheme of apical constriction. **b** Design of OptoShroom3 constructs that dimerize upon blue light stimulation. **c** MDCK cells expressing GFP-NShroom3-iLID and SspB-mCherry-CShroom3 before and after 1-min stimulation. Top: x-y apical slice, bottom: x-z lateral slice. Scale bar = 10 μm. **d** Non-muscle myosin IIb stainings of OptoShroom3 MDCK cells under no stimulation (left) and 2-h blue light stimulation (right). Apical slices. Scale bar = 10 μm. **e** Single-cell stimulation cycles and representative images from constriction experiments. Apical slice. Stimulated area was designed as the initial apical area of the cell. iRFP-CAAX signal. Right panel shows a color-coded compounded image comparing the start and end of stimulation. Scale bar = 10 μm. **f**, **g** Quantification of the apical area in stimulated (*N* = 17) and non-stimulated (*N* = 16) cells. The area was normalized to the last measurement before stimulation (*t* = 5 min). Avg ± sd. **h** Quantification of apical and basal areas during 3 periods of 10 min of stimulation and rest (*N* = 8, avg ± sd). The basal slice was defined as ~5 μm below the apical slice. The areas were normalized to the last measurement before stimulation (*t* = 10 min).

To achieve spatiotemporal control over apical constriction in mammalian tissues, we created an optogenetic version of Shroom3. Shroom3 causes apical constriction by recruiting ROCK to apical junctions^31^. The Shroom Domain 1 (SD1) of Shroom3 is an actin-binding motif responsible for the apical localization^26,32,33^, whereas the SD2 is necessary for the binding to ROCK^31^ (Fig. 1a). The SD1 and SD2 domains are shown to function independently^26,34^. Therefore, we hypothesized that these domains could be split into two constructs and that the protein functionality could be restored through light-induced binding of the iLID-SspB optogenetic pair^35^. Upon blue light illumination, iLID will change conformation and make its binding site accessible for SspB. After testing multiple domain combinations, we found that the N-terminal Shroom3 fused with iLID (hereafter called NShroom3-iLID) and the C-terminal Shroom3 fused with SspB (SspB-CShroom3) function as an optogenetic split-version of Shroom3 (OptoShroom3) (Fig. 1b). GFP-NShroom3-iLID localized similarly to Shroom3 (Fig. 1c left; Supplementary Fig. 1) to the apical junctions of MDCK cells. By contrast, SspB-mCherry-CShroom3 acquired apical localization upon blue light illumination (Fig. 1c right).

We measured the translocation dynamics of Sspb-mCherry-CShroom3 in MDCK cells stably expressing both OptoShroom3 constructs, showing a 1.75-fold increase in apical junctional signal within seconds after blue light illumination (Supplementary Fig. 2). Once stimulation ended, it required 100 s for the junctional signal to return to the off state. OptoShroom3 unbinding half-life was ~30 s. Having SspB instead of SspB-CShroom3 did not alter the on-off translocation rates, suggesting that these dynamics are due to the intrinsic properties of the iLID-SspB pair and not affected by interactions of CShroom3 with potential partners in the apical junctions (Supplementary Fig. 2a, b, GFP-NShroom3-iLID + SspB-mCherry (MDCK)). These results are consistent with previous reports of the half-life of iLID-SspB binding, which is <1 min^35^. In addition, a non-binding OptoShroom3 construct was built based on a LOV domain mutation (C450V) in NShroom3-iLID that prevents iLID from changing conformation and binding to SspB^36^. The mutant variant (hereafter called C450V mutant) did not show the translocation of Sspb-mCherry-CShroom3 upon illumination (Supplementary Fig. 2a, b).

To test the ability of OptoShroom3 to induce apical constriction, OptoShroom3 MDCK cells were stimulated with blue light for 2 h and fixed for immuno-fluorescence staining. The photostimulated cells showed straight apical junctions (Supplementary Fig. 3a) and zipper structures of non-muscle myosin IIb in the apical junctions similar to those that had been previously described for wild-type Shroom3^34^ (Fig. 1d; Supplementary Fig. 3b, c). The stimulated cells did not show qualitative changes in F-actin or ɑ-tubulin distribution (Supplementary Fig. 3d, e). These results suggest that OptoShroom3 induces apical constriction by causing a redistribution of non-muscle myosin II in the apical junctions.

To study the dynamics of OptoShroom3-induced apical constriction, we stimulated a single cell in an MDCK monolayer expressing OptoShroom3 (Fig. 1e). The 1-min illumination cycle was designed by taking into account the measured binding dynamics of OptoShroom3. The apical surface area showed a rapid decrease during the first minutes of stimulation, and constriction gradually decelerated, achieving a 25.4 ± 8.9% reduction of the original area within 50 min (Fig. 1e–g; Supplementary movie 1). The apical area started to increase only 1 min after the end of stimulation, matching the fast unbinding of the two components and pointing out that the deactivation of actomyosin contraction is equally fast (Fig. 1f). These constriction dynamics are similar to those observed by Cavanaugh et al. using RhoGEF recruitment for cell-junction shortening in mammalian cells^20^. We then tested whether OptoShroom3 could be repeatedly activated and deactivated by concatenating several stimulation-rest periods (Fig. 1h; Supplementary movie 2). The apical area measurement indeed displayed periodic constrictions and fast activation-deactivation responses (<1 min each). By contrast, the basal area did not show constriction upon stimulation but displayed a slightly increasing tendency during the stimulation phases. We reasoned that the subtle increase on the basal side could be due to membrane and cytoplasmic influx from the apical side, similarly to what has been described during *Drosophila* ventral furrow invagination^37^. These measurements collectively demonstrate that OptoShroom3 effectively reduces the apical-basal ratio of a stimulated cell (Supplementary Fig. 4) and has fast activation and deactivation kinetics.

### Induction of apical constriction increased apicobasal length

**Fig. 2:**
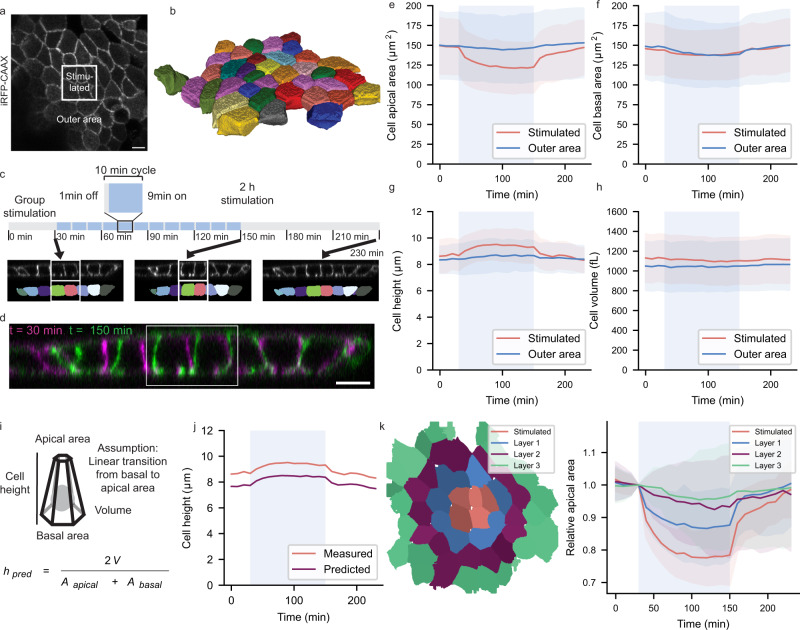
OptoShroom3-induced cell elongation. **a** Apical slice of iRFP-CAAX signal of MDCK cells on glass. The white box marks the stimulated area (26.5 × 26.5 μm). Scale bar = 10 μm. **b** Reconstruction of a 3D segmented MDCK monolayer. **c** Cell group stimulation cycles and representative images from constriction experiments. Lateral slice of iRFPCAAX and 3D segmentation. **d** Color-coded compounded image comparing the start and end of stimulation. Scale bar = 10 μm. **e**–**h** Apical, basal surface, cell height, and volume measurements for 2 h stimulation of OptoShroom3 MDCK cells, separating between stimulated cells and the rest of the cells in the frame (outer area) (*N*_Experiments_ = 5, *N*_Stimulated_ = 61, *N*_Outer_ = 137, avg ± sd). **i**, **j** Model prediction of cell height based on average measurements shown in panel **g**. **k** Re-analysis of apical area reduction classifying cells by layers (*N*_Experiments_ = 5, *N*_Stimulated_ = 20, *N*_Layer1_ =  51, *N*_Layer2_ = 88, *N*_Layer3_ = 44, avg ± sd). Reanalysis of Fig. 2e.

To further investigate the effects of OptoShroom3 activation on cell shape, timelapses of plasma membrane-labeled OptoShroom3 MDCK cells were acquired for 3D segmentation. A rectangular area (average 4 cells, Fig. 2a) was stimulated for 2 h or 20 min (Fig. 2a–c; Supplementary Fig. 5a, b). The cells stimulated for both 20 min and 2 h underwent clear and reversible apical constriction (Fig. 2d, e; Supplementary Fig. 5a, b). Differently from what we observed for single-cell stimulation, the basal area did not undergo any clear changes during group cell stimulation (Fig. 2f). It could be that, when a group of cells is stimulated, basal areas cannot expand simultaneously due to a lack of space. By contrast, we observed an average 10% increase in cell height along the apical-basal axis in the stimulated cells (Fig. 2g; Supplementary movie 3), which was reversed after the end of stimulation. Cell volume remained constant in all cases (Fig. 2h). OptoShroom3 C450V mutant did not undergo apical constriction or cell elongation (Supplementary Fig. 5c, d). In addition, the reduction of apical area provoked by OptoShroom3 stimulation was similar but not as strong as the area reduction induced by sparse Shroom3 overexpression (Supplementary Fig. 6a, b). We concluded that OptoShroom3 induces reversible apical constriction and cell elongation in confluent MDCK monolayers while maintaining the cell volume and basal area (Supplementary Fig. 5d).

With the aim of gaining more understanding of the cell elongation mechanism, a simple model assuming a linear change in cross-sectional area from basal to apical was used (Fig. 2i; Supplementary Fig. 7a). By introducing measured volumes, apical, and basal areas, we successfully predicted the cell height dynamics (Fig. 2j). The same predictions were carried out for the other parameters (Supplementary Fig. 7b). For all cases, predictions accurately matched the measured dynamics while being offset from the real values. The offset could be due to an underestimation in volume measurements. Still, these results point out that, given a reduction in apical area, an increase in cell height can be expected when volume and basal area are conserved.

To improve our understanding of the effect of OptoShroom3 activation on the cells surrounding the stimulated area, we made a classification by layers, layer 1 including the cells directly in contact with the stimulated cells, layer 2 being the cells in contact with the cells in layer 1, and so on (Fig. 2k). All the layers present in the imaging frame (stimulated, layer 1, layer 2, and layer 3) showed a reduction in apical area, less pronounced the further away from the stimulated area (Fig. 2k). This could be due to light diffusion weakly activating the layers surrounding the illumination area. As no layer of cells displayed expansion of the apical area, we hypothesized that OptoShroom3 may provoke cell displacements to accommodate the gradient of apical constriction.

### OptoShroom3 activation caused displacement of adjacent cells

**Fig. 3:**
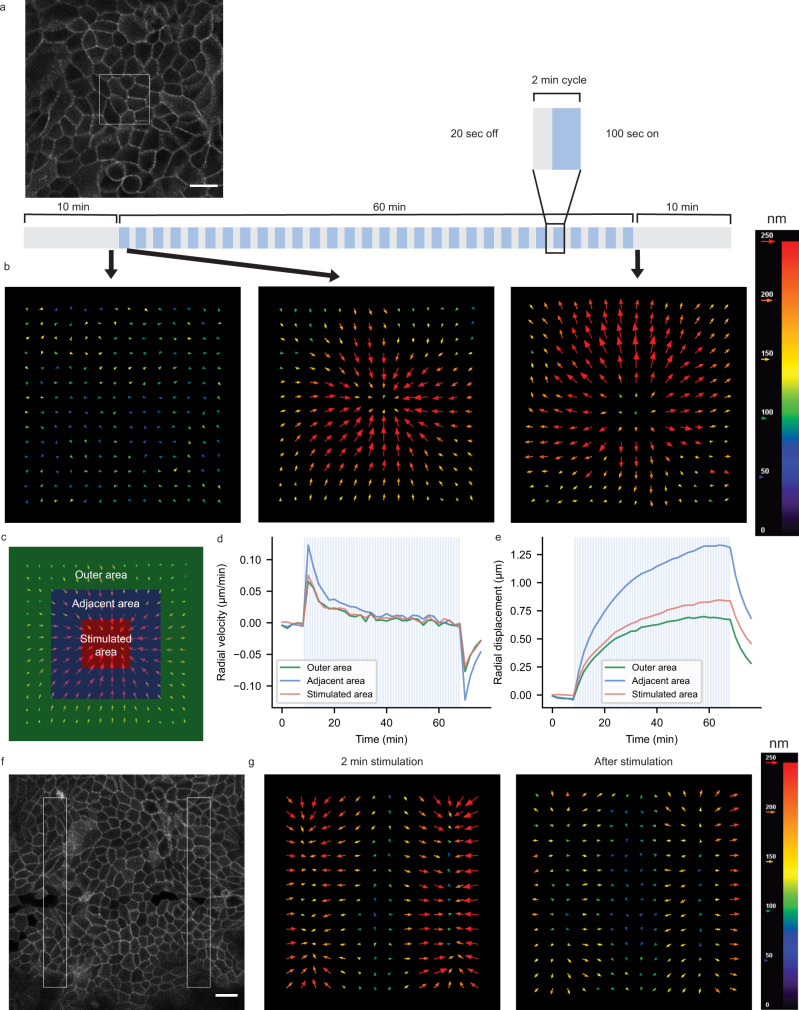
OptoShroom3-induced apical displacements on confluent monolayers. **a** Apical slice of iRFP-CAAX signal of MDCK cells on a thick collagen gel. The white box marks the stimulated area. Scale bar = 20 μm. **b** Averaged PIV analysis on apical slices before, during (2 min), and after stimulation in a 35.3 × 35.3 μm area (*N* = 6). **c** Division of PIV frame in three areas for analysis, stimulated, adjacent, and outer areas. **d** Temporal evolution of radial vector components averaged by area. Positive vectors represent movements towards the center of stimulation (*N* = 6, avg). **e** Integration of vectors averaged by area (*N* = 6, avg). **f** Apical slice of iRFP-CAAX signal of MDCK cells on a collagen gel. The white rectangles mark two stimulated areas. Scale bar = 20 μm. **g** Averaged PIV analysis on apical slice during (2 min) and after stimulation of two 20 × 170 μm areas (*N* = 7).

To study potential displacements provoked by OptoShroom3 in the cells surrounding the stimulated area, we reasoned that a setting softer than glass could allow for clearer cell movements. Therefore, confluent MDCK monolayers were formed on thick collagen gels. We found that sustained stimulation of a group of cells inside an illumination square (average 12 cells, Fig. 3a) caused cellular displacements towards the stimulated area on the apical side, whereas displacements in the opposite direction were observed after the end of stimulation (Supplementary movie 4). Particle image velocimetry (PIV) analyses on the apical slices displayed a peak in inward displacements 2 min after stimulation, which gradually disappeared within the next 1 h (Fig. 3b–d). The second peak of similar magnitude was observed in the outward direction immediately after the stimulation ended (Fig. 3d). Vectors showing the highest velocity, both upon start and end of stimulation, were those adjacent to the stimulated area (Fig. 3d, adjacent area). The accumulated displacement (summation of measured vectors over time) accounted for 1.31 ± 0.63 μm in the adjacent area (Fig. 3e). This result indicates that OptoShroom3 causes the largest displacement at the border between constricting cells and non-stimulated cells. Similar but smaller displacements were measured for the stimulated and outer area (0.84 ± 0.37 and 0.67 ± 0.29 μm, respectively). The highest displacement was achieved at 0–5 μm from the stimulated area, and displacement was observed up to 60 μm away from the stimulated area (Supplementary Fig. 8). Next, we produced a more complex deformation pattern through the simultaneous stimulation of two areas (Fig. 3f; Supplementary movie 5). PIV analyses visualized tissue displacements with the shape of the stimulated areas (Fig. 3g). These results demonstrate that OptoShroom3 can induce 2D tissue-level displacements by pulling the apical surface of cells that are adjacent to stimulated areas.

### Illumination of OptoShroom3 cell colonies induced folding

**Fig. 4:**
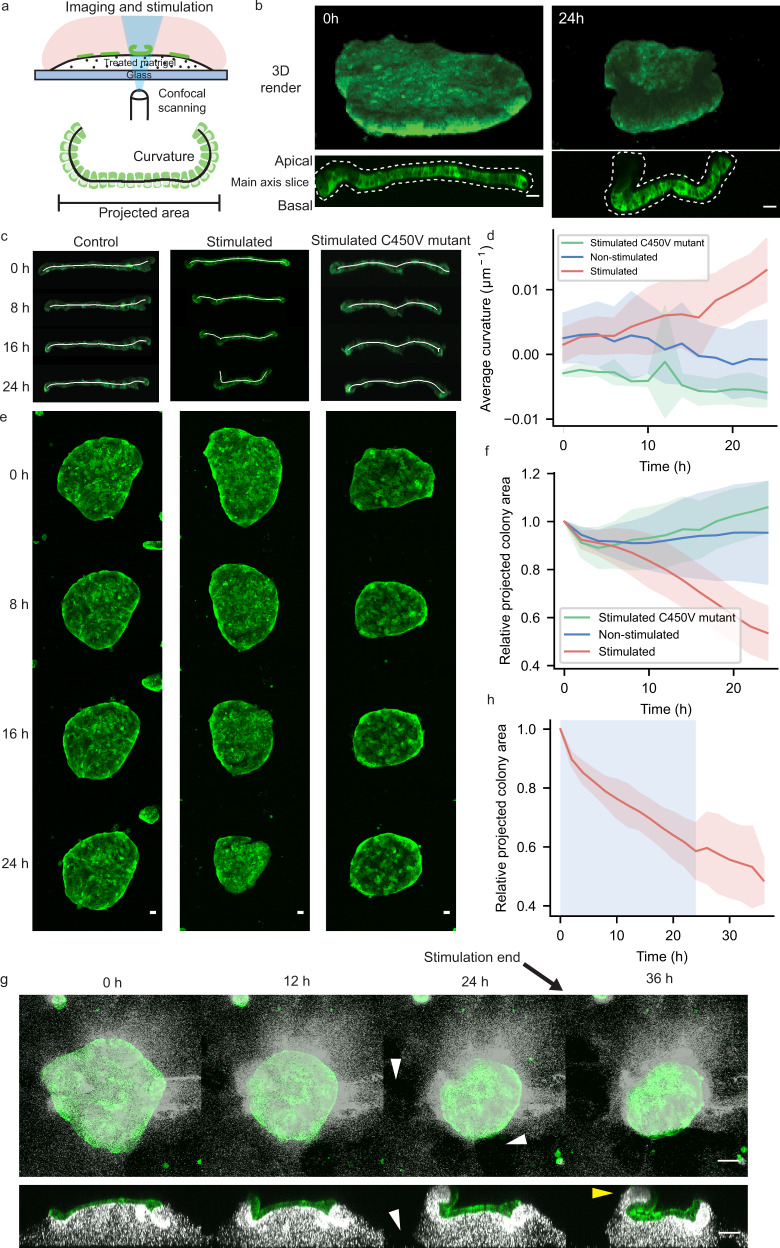
OptoShroom3-induced colony folding. **a** Protocol for the formation of flat MDCK monolayers in a thick matrigel bed using acetic acid incubation (top) and scheme of measured parameters (bottom). **b** 3D renders and higher resolution lateral slices of representative colony folding before and after 24 h of stimulation (stimulated cycle: 3 min off, 57 min on; non-stimulated cycle: 3 min on (GFP-NShroom3-iLID image acquisition), 57 min off). GFP-NShroom3-iLID expression. Scale bar = 20 μm. **c** Skeletons used for curvature measurements of representative examples of non-stimulated (left), stimulated (center), and stimulated C450V mutant (right) colonies. GFP-NShroom3-iLID expression. **d** Quantification of changes in average curvature of stimulated, stimulated C450V mutant and non-stimulated colonies. Concave (apical) curvature was set as positive (*N*_Stimulated_  = 7, *N*_Non-stimulated_ = 7, *N*_C450Vmutant_ = 5, avg ± sd). **e** Z-projected images of colonies used for area measurements of non-stimulated (left), stimulated (center), and stimulated C450V mutant (right) colonies. GFP-NShroom3-iLID expression. Scale bar = 20 μm. **f** Quantification of relative changes in the projected area of stimulated, stimulated C450V mutant, and non-stimulated colonies (*N*_Stimulated_ = 7, *N*_Non-stimulated_ = 7, *N*_C450Vmutant_ = 5, avg ± sd). **g** Representative example of irreversible colony folding on matrigel labeled with infra-red fluorescent beads. White arrowheads indicate matrigel breaking points, and yellow arrowhead indicates matrigel being deformed and carried over with the folding colony. Scale bar = 20 μm. **h** Quantification of relative changes in the projected area of stimulated colonies during and after stimulation (stimulation period in blue) (*N* = 5, avg ± sd).

To further assess if OptoShroom3 can induce 3D tissue morphogenesis in MDCK cells, we used matrigel, which is a well-known soft deformable gel. However, MDCK cells did not form a flat monolayer on a thick matrigel bed. We found that treatment of matrigel with acetic acid enabled the formation of flat MDCK monolayer colonies by increasing gel stiffness (Supplementary Fig. 9). Stimulation of a whole colony in this setting caused folding of the MDCK cell sheet in 24 h (Fig. 4a, b; Supplementary movie 6). This time course resembles those of tissue folding processes in mammalian development, such as neural tube- and gut-folding, which require 1 or more days depending on the species^38–40^. Measurements of colony skeletons from x-z sections displayed an increase in average curvatures of stimulated colonies, compared with the unchanged curvatures of non-stimulated colonies and OptoShroom3 C450V mutant colonies (Fig. 4c, d). The curvature did not homogeneously increase in folding colonies, and the peripheral cells were displaced first towards the center of the colony (Fig. 4c; Supplementary Fig. 10). Consistent with the increase in curvature, the projected area of stimulated colonies showed a decrease upon stimulation, reflecting the induced contractility (Fig. 4e, f). Stimulated OptoShroom3 C450V mutant colonies and non-stimulated OptoShroom3 colonies did not fold. These measurements suggest that apical constriction changes the cell shape to pyramidal, inducing upward folding of a monolayer colony on a soft gel.

To test the reversibility of tissue folding and visualize substrate deformation, we added infra-red fluorescent beads to matrigel and recorded the colony morphology even after 24-h stimulation (Fig. 4g). When stimulation was over, the folding of OptoShroom3 MDCK colonies kept on progressing in the following hours (Fig. 4h; Supplementary movie 7). Fluorescent beads allowed us to observe how folding was achieved through deformation and breakage of the gel, instead of tissue detachment (Fig. 4g). To further test the controllability of the cell-sheet folding, we chose colonies with elongated shapes and stimulated a restricted area of a cell colony. Although the success of this approach depended on the initial shape of the colony, the spatial illumination led to the coiling and retraction of the stimulated area (Supplementary Fig. 11; Supplementary movie 8). These results show that OptoShroom3 can irreversibly provoke tissue curvatures and folds in a spatiotemporal manner and that tissue folding is achieved through deformation and remodeling of matrigel.

### OptoShroom3 induced deformation in neural organoids

**Fig. 5:**
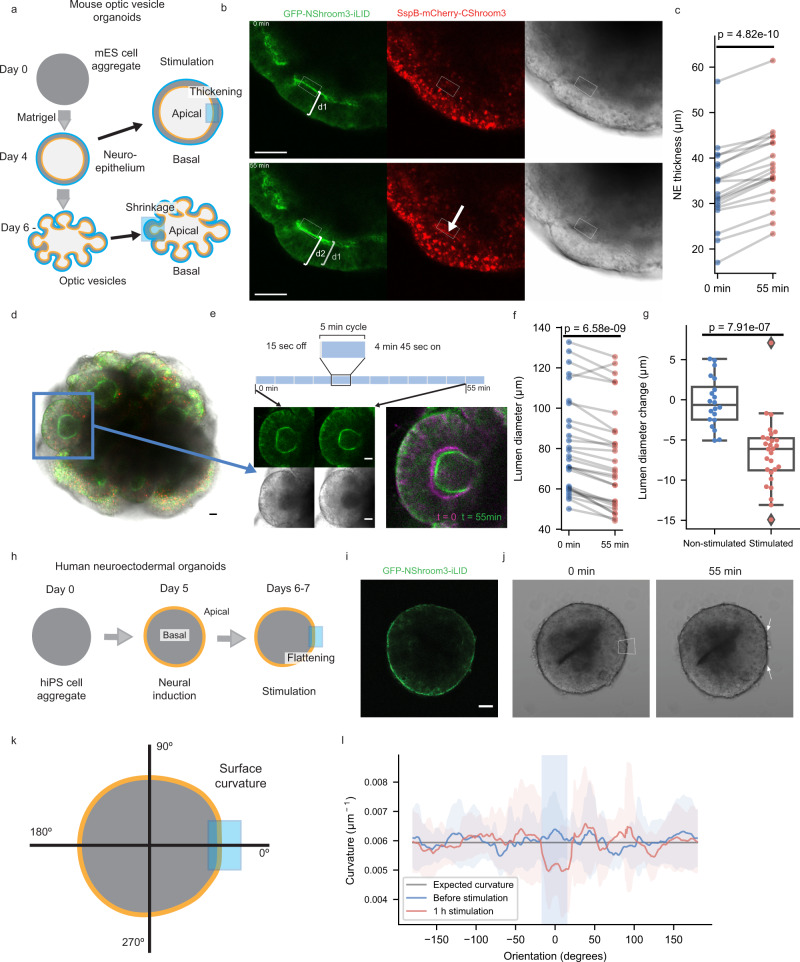
OptoShroom3-induced tissue deformation in mouse and human neural organoids. **a** The formation and stages of mouse optic vesicle organoids. **b** Representative example of stimulation of neuroepithelium (day 5, single plane). Arrow indicates the translocation of SspB-mCherry-CShroom3. Scale bar = 50 μm. **c** Thickness measurements of neuroepithelia before and after stimulation (days 4–8) (*N* = 19, paired *t*-test, two sided). **d** Representative example of optic vesicle organoid on day 8. Scale bar = 20 μm. **e** Stimulation cycles and representative example of 488 nm stimulation of an optic vesicle showing transmitted light and GFP-NShroom3-iLID signal. Right panel shows a color-coded compounded image comparing the start and end of stimulation. Scale bar = 20 μm. **f** Measurements of lumen diameter of optic vesicles before and after OptoShroom3 stimulation (days 6–8) (*N* = 27, paired *t*-test, two sided). **g** Quantification of the lumen diameter change for control (non-stimulated) and stimulated samples after 55 min (*N*_Non-stimulated_ = 17, *N*_Stimulated_ = 27, student’s *t*-test, two sided). Boxplot boundaries first to third quartile. Central line: median. Whiskers: 1.5x the inter-quartile range. Min and max are displayed by the dotplot. **h** The formation stages of human neuroectodermal organoids. **i** GFP-NShroom3-iLID expression on a neuroectodermal organoid on day 6. Scale bar = 50 μm. **j** Representative example of OptoShroom3-induced flattening through local stimulation of neuroectodermal organoids. **k** Schematic of curvature analysis. The center of the stimulated area was set up as 0 degrees. **l** Curvature measurements of neuroectodermal organoids before and after stimulation (*N* = 8, avg ± sd).

To alter mammalian tissue structure in more complex systems, we further tested the application of OptoShroom3 in mouse optic vesicle organoids. Here we denominate optic vesicle organoids to the earlier stages of optic cup organoids^41^, which are known to develop polarized neuroepithelia that form optic vesicles. Optic cups have been demonstrated to form through the up-regulation and down-regulation of apical constriction^41,42^. Towards this aim, we prepared a mouse embryonic stem (mES) cell line stably expressing OptoShroom3 and created optic vesicle organoids (Fig. 5a). GFP-NShroom3-iLID changed its localization along with organoid differentiation, from a diffused signal (days 1–3) to a concentrated signal on the apical side of the neuroepithelium (day 4–) (Supplementary Fig. 12). It is important to mention that the inner surface of optic vesicle organoids is apical. We considered the change in localization an indication of the proper differentiation of the neuroepithelium. Stimulation of OptoShroom3 in the earlier stages of a neuroepithelium (days 4–8) caused translocation of SspB-mCherry-CShroom3 with similar dynamics to those of MDCK cells (Supplementary Fig. 2c, d). Stimulation also caused a 14.3 ± 7.4% increase in the thickness of the epithelial layer (Fig. 5b, c; Supplementary movie 9). The stimulation of a whole optic vesicle (days 6–8) caused a decrease in the size of the apical lumen (Fig. 5d, e; Supplementary movie 10). The longest diameter of the lumen displayed a 9 ± 5.8% decrease after ~1 h of continuous stimulation (Fig. 5f, g). It had been previously shown that Shroom3 could induce cell elongation through the induction of microtubule polymerization along the apical-basal axis through redistribution of γ-tubulin^43^. To test this hypothesis, we visualized microtubules with SiR-tubulin and repeated the neuroepithelium stimulation experiment. Microtubules were already polymerized along the apical-basal axis in optic vesicles even before stimulation, and OptoShroom3 activation did not seem to provoke polymerization of new microtubules (Supplementary movie 11). This result suggests that the observed thickening is a mechanical process, similar to the cell elongation observed in MDCK cells (Fig. 2). We reasoned that apical constriction made individual cells taller, effectively reducing the apical lumen size.

To test if the reversed apicobasal polarity leads to a different type of tissue deformation, we utilized human neuroectodermal organoids. Neuroectodermal organoids were made from human-induced pluripotent stem (hiPS) cells stably expressing OptoShroom3, following the first steps of cerebral organoid formation^44^ (Fig. 5h). Contrary to optic vesicle organoids, neuroectodermal organoids show outer apical polarity before matrigel addition and therefore exhibit a convex apical side. Accordingly, we observed that GFP-NShroom3-iLID localized on the outer surface of the organoid upon neural induction (days 6–7) (Fig. 5i). Selective stimulation of a border of neuroectodermal organoids provoked flattening of the area, reducing the curvature by 18.5 ± 1.5% in 1 h (Fig. 5j–l) and displaying inward tissue displacement (Supplementary movie 12). These results demonstrate that OptoShroom3 can manipulate morphologies of organoids, including epithelial thickening, lumen reduction, and flattening. The outcome of deformation depends on initial tissue geometry, apicobasal polarity, and the forces to which the tissue is already subjected to.

## Discussion

In this study, we have developed an optogenetic tool to control apical constriction in mammalian tissues. The tool induced a quick reduction of the apical cell surface (starting 1 min after stimulation), and the stimulation of groups of cells provoked cell elongation and collective displacements in the apical surface of adjacent cells. Apical constriction in epithelial colonies on soft gels induced the irreversible folding of cell sheets. In organoids, apical constriction thickened the neuroepithelium and reduced the inner lumen when the inner surface was apical. By contrast, it flattened the tissue when the outer surface of organoids was apical. These results illustrate how the induction of apical constriction in different contexts can lead to different changes in tissue structure.

A possible explanation for these differences is the specific tissue geometry and availability of cytoskeletal components of each cell type, as well as the interactions with the extracellular matrix. According to our results, OptoShroom3 activation provokes the recruitment of non-muscle myosin IIb to the apical junctions. Although it has been reported that Shroom3 can induce a redistribution of γ-tubulin to cause cell elongation along the apicobasal axis^43^, we did not find a change in the distribution of microtubules in MDCK cells or optic vesicle organoids upon OptoShroom3 activation. Moreover, our results on 3D cell segmentation and the model suggest that OptoShroom3-induced apical constriction could itself be enough to explain cell elongation through cell volume conservation, consistent with previous studies^45,46^. The presented experiments support the idea that OptoShroom3 induces actomyosin constriction specifically on the apical side, and that cell elongation is a consequence of the induced apical constriction.

OptoShroom3 joins the expanding set of optogenetic tools capable of inducing actomyosin constriction. The speed and constriction rate of OptoShroom3 are similar to the results presented by Cavanaugh et al. for optogenetically activated RhoA in cell junction shortening^20^. However, Cavanaugh et al. reported that stimulation longer than 5 min caused an irreversible reduction of cell junctions. OptoShroom3-induced apical constriction and cell elongation in MDCK cells on glass were reversible independently of the duration of the stimulation. By contrast, OptoShroom3-induced colony folding was irreversible and provoked changes in the matrigel substrate. These results suggest that when OptoShroom3-induced force can be transduced to a deformable substrate, permanent changes can be achieved.

The main advantage of OptoShroom3 lies in its specificity. Pre-existing tools have mainly used plasma membrane recruitment of RhoGEF or RhoA factors for the activation of actomyosin constriction^18–22^. By contrast, the recruitment of OptoShroom3 is specific to the apical junctions of epithelial cells because of the specific and continuous localization of GFP-NShroom3-iLID. Therefore, OptoShroom3 does not require subcellular precision of stimulation by multi-photon microscopy to induce apical constriction. Even if a whole cell is stimulated by a scanning laser or an LED, SspB-mCherry-CShroom3 will be recruited to the apical side and induce constriction in that area only. This is advantageous especially to stimulate tissue with complex shapes, such as the apical surface of an optic vesicle. By contrast, OptoShroom3 cannot induce actomyosin constriction in basal or lateral areas or in cells that do not possess apicobasal polarity.

The rapid activation-deactivation cycle of OptoShroom3 can be an advantage when studying dynamic and fast processes. Although this may be considered a limitation when planning long-term experiments, continuous stimulation did not cause visible phototoxicity in the 24-h folding experiments. A potential way to reduce stimulation periods would be to take advantage of mutant versions of LOV2 domain of iLID known to increase the half-life of its binding to SspB^36,47^. These features highlight OptoShroom3 as a tool to externally manipulate and control tissue shapes and to study tissue mechanics^48–50^ and mechanotransduction^51,52^.

In the long term, novel tools that can induce morphological changes will be necessary not only to study in vivo morphogenesis but also to reproduce the morphogenetic processes in vitro. We expect that OptoShroom3 will provide a new avenue towards understanding how tissue shape and mechanotransduction affect cell fate and differentiation. Organoids, given their inherent complexity and accessibility, stand out as perfect candidates for the application of these new morphogenetic tools to further study the interplay between tissue shape and function.

## Methods

### Cloning and construction of optogenetic tool

For OptoShroom3 design, mouse *Shroom3* gene was used, which was a gift from T. Nishimura from M. Takeichi lab. Shroom3b isoform (originally named ShrmS^26^), which presents an N-terminal deletion of 177 aa that removes a PDZ domain, was used for the construction. Construction of OptoShroom3 was carried out using iLID and Sspb genes from the optogenetic vector library published by Tichy et al.^53^, which was a gift from H. Janovjack lab. OptoShroom3 and OptoShroom3 C450V mutant sequences can be found in Supplementary Table 1. OptoShroom3 constructs GFP-NShroom3-iLID and SspB-mCherry-CShroom3 have been submitted to Addgene (catalog numbers: 170976 and 170977, respectively). iRFP-CAAX and GFP-CAAX sequences were prepared through traditional cloning methods. iRFP gene was acquired from Addgene (piRFP, plasmid #31857). For stable construct expression, CAG promoter was used for OptoShroom3 and GFP-CAAX, and Ef1α promoter was used for iRFP-CAAX.

### Cell culture

MDCK cell line (MDCKII) was a gift from M. Murata lab, which was previously obtained from Dr. Kai Simons lab. MDCK cells were maintained in Dulbecco’s Minimal Essential Medium (DMEM) with high glucose, GlutaMax, and pyruvate (Gibco, 31966-021), supplemented with 10% fetal bovine serum, penicillin, and streptomycin (100 U/ml and 100 μg/ml, Gibco). Cells were split every 2–3 days through 9-min incubation with EDTA 50 mM pH = 7.4 and 3 min with 0.25% trypsin (Gibco).

Mouse ES cell line (EB5, Rx-GFP) was described previously^41^. mES cells were maintained on gelatin-coated dishes with DMEM supplemented with 15% fetal bovine serum, nonessential amino acids, GlutaMax, sodium pyruvate (Gibco, 11360-039), β-mercaptoethanol (0.1 mM), LIF (1500 U/ml), CHIR99021 (3 µM), and PD0325901 (1 µM). The 2i medium was used to increase the number of optic vesicles per organoid. Cells were split every 2–3 days with 0.25% trypsin.

Human iPS cell line ((IMR90)−4) was purchased from WiCell. hiPS cells were maintained in StemFlex media (Gibco) on matrigel-coated dishes. DMEM-F12 (Gibco) with 1.23% matrigel (Corning #356231) was used for coating for 30 min at 37 °C. Cells were split every 4–5 days with Accutase (STEMCELL Technologies) and StemFlex media was supplemented with ROCK inhibitor Y-27632 (10 µM) during the first day after splitting. Since using publicly available human iPS cells constitutes acceptable practice in EMBL, the ethics evaluation of the project was officially exempted by the institute.

For MDCK and mES cells, OptoShroom3, OptoShroom3 C450V mutant, iRFP-CAAX, and GFP-CAAX constructs were stably transfected using PiggyBac system^54^ and lipofectamine. For hiPS cells, PiggyBac plasmids were introduced using Amaxa Nucleofector (Lonza). After antibiotic selection, stable clones were picked for every cell line. All cell lines were cultured at 37 °C in a humidified incubator with 5% CO_2_ and regularly tested for mycoplasma contamination.

### Live imaging and stimulation of optogenetic tool

All live imaging was acquired using Olympus FV3000 confocal microscope at 37 °C and 5% CO_2_. Stimulation of OptoShroom3 in all samples was performed using stimulation mode and 0.01–0.03% power of 488 nm laser. UPLSAPO40XS and UPLSAPO30XS objectives were used for MDCK imaging and stimulation, and UPLSAPO10X2 was used for organoid imaging and stimulation. Only Supplementary movie 11 was acquired on Leica Stellaris confocal microscope (using software Leica Stellaris Las X) at 37 °C and 5% CO_2_.

### Immuno-fluorescence staining

MDCK samples for immuno-fluorescence staining were prepared by seeding 2 × 10^5^ MDCK cells on a 12 mm diameter glass (113 cells/mm^2^). Whole sample stimulation was carried out 2 days after seeding with an array of blue LEDs inside a cell-culture incubator. Samples were placed on top of the LED array with a white methacrylate diffuser to make light homogeneous. After 2 h of darkness or stimulation, cells were fixed at 37 °C and 5% CO_2_ maintaining the stimulation or darkness condition. Primary and secondary antibody incubations were carried out for 1 h at room temperature. Antibodies and probes used were: rabbit Non-muscle Myosin Heavy Chain II-B Antibody (dilution 1:200, Biolegend, 909902), anti-α-tubulin mouse mAb (dilution 1:400, Sigma, DM1A), phalloidin-atto 655 (dilution 1:200, Sigma), alexa fluor 647 goat anti-rabbit (dilution 1:200, Invitrogen), and alexa fluor 647 goat anti-mouse (dilution 1:200, Invitrogen).

### Image analysis

Apical and basal area measurements in Fig. 1 were conducted on a custom-made python pipeline, applying watershed segmentation to iRFP-CAAX confocal stacks of MDCK cells on glass-bottom dishes. Basal stacks were selected as ~5 µm below apical. Projected area and skeletons for curvature measurements of folding MDCK colonies were obtained from confocal stacks using a custom-made python segmentation pipeline.

For the analysis of non-muscle myosin IIb intensity in immunofluorescence stainings, the GFP-NShroom3-iLID signal was used to segment the apical junctions. Then, the average intensity per pixel of non-muscle myosin IIb in the junctional area was divided by the average intensity per pixel in the cytoplasmic area (considered as the remaining pixels in the image).

3D segmentation was carried out with a custom pipeline using 3D timelapses obtained with 0.207 μm/pixel x-y resolution and 0.7 μm/pixel z-resolution. The stacks were then rescaled to 1:1:1 voxel dimension (0.207 μm/pixel) through linear interpolation before segmentation. The segmentation protocol was validated through comparison with the segmentation of 80 cells in MorphoGraphX^55^. In both cases, the measurements showed minimal differences, with the advantage of our custom pipeline being fully automated.

Cell volume was calculated through voxel counting of the segmented cells. Cell height was measured by finding the distances between the average height of contact with the apical and the basal side. Apical and basal areas were measured through the acquisition of a 2D image of the cell voxels in contact with either the apical or basal side. Cells were tracked from frame to frame based on maximum overlap. For all measurements, quality control of the segmentation was carried out by discarding cells that were not present in all frames of the timelapse. Cells that had sudden changes in volume higher than 30% (mis-segmented, dividing and apoptotic cells) were also eliminated from the analysis.

For the classification shown in Fig. 2e–h and Supplementary Fig. 5, all cells in contact with the stimulation area were considered as stimulated. For the layer classification, only cells with more than 50% of their apical area inside the illumination area at the start of the stimulation were considered stimulated.

### Translocation measurement

For the analysis of translocation, 2 × 10^5^ MDCK cells were seeded on a 12 mm-diameter glass (113 cells/mm^2^). Samples were stimulated 2 days after seeding as described in live imaging methods. Translocation measurements were carried out with FIJI using apical slices of MDCK cells. A 0.362 μm^2^ area (5 × 5 pixels) of the junctional or cytoplasmic region was manually selected and averaged for every time point. Three areas for each type (junction or cytoplasm) were selected on every image. For the junction/cytoplasm ratio, each junction measurement was divided by the closest cytoplasm measurement.

### Sample preparation for 3D segmentation

For the analysis cell shape, 2 × 10^5^ MDCK cells were seeded on a 12 mm-diameter glass (113 cells/mm^2^). Samples were stimulated 2 days after seeding as described in live imaging methods. For Shroom3 measurements, a stable GFP-CAAX and doxycycline-inducible mCherry clonal cell lines were mixed at 5% ratio with a GFP-CAAX stable cell line. Samples were induced with 0.5 μg/ml 24 h before the measurement. For WT-control measurements, only the GFP-CAAX stable cell line was used.

### Curvature measurement

Local curvature was defined as the inverse of the radius of the osculating circle to three points of a curve. Curvature was measured by calculating the osculating circle of three equally distanced points (20 pixels distance) in the skeleton, equivalent to calculating the inverse of the radius of a circumscribed circle around a triangle:

$${{{{{\rm{Curvature}}}}}}=\frac{1}{R}=\frac{4K}{{{{{{\rm{AB}}}}}}\cdot {{{{{\rm{BC}}}}}}\cdot {{{{{\rm{CA}}}}}}}$$

Being *R* the radius of the osculating circle of points A, B, and C. AB, BC, and CA are the distance between the points. *K* is the area of the triangle formed by A, B, and C. Local curvatures were measured along the skeleton of the main axis and averaged for every time point to obtain a general curvature measurement.

For neuroectodermal organoids, SspB-mCherry-CShroom3 slices were segmented. Curvature measurements were applied to the perimeter of the segmented organoids. In this case, distance between points was set up higher (320 pixels) to filter out curvatures caused by very small features, such as delaminating cells.

### PIV analysis

Particle Image Velocimetry analyses were conducted on iRFP-CAAX projections of apical slices of MDCK cells seeded in 100% collagen gels. For preparation, 50 μl of 100% collagen I were added to a glass-bottom dish and incubated at 37 °C for 30 min for polymerization. After incubation, 5 × 10^5^ OptoShroom3 + iRFP-CAAX MDCK cells were seeded in 80 μl of DMEM medium. More media (2 ml) was added 1.5 h after seeding. Confluent monolayers were stimulated 2 days after seeding using 0.03% power of 488 nm laser. Images were acquired every 2 min. PIV analysis was performed using the ImageJ PIV analysis plugin developed by Q. Tseng^56^ (https://sites.google.com/site/qingzongtseng/piv). The cross-correlation method was used with 1 pass with an interrogation window size of 64 pixels. Batch analysis, classification of vectors by area, and experiment averaging were performed with a custom python pipeline.

### Tissue folding

For MDCK colony folding assays, 20 μl of 100% matrigel (Corning #356234) were set on glass-bottom dishes and solidified at 37 °C for 10 min. Then, gels were incubated with 20 mM acetic acid (90 μl, 4.5 matrigel-acetic acid volume ratio) at 37 °C for 40 min. After incubation, gels were washed twice with PBS and 4 × 10^5^ MDCK cells were seeded in 80 μl of DMEM medium. More media (2 ml) was added 1.5 h after seeding. These parameters were empirically obtained to make isolated flat MDCK colonies, which were stimulated 2 days after seeding.

Stimulated colonies were illuminated for 57 min every hour. The remaining 3 min were used for the acquisition of GFP-NShroom3-iLID signal in control colonies (non-stimulated colonies). We considered the impact of this period of illumination on control colonies and of lack of illumination on stimulated colonies negligible. Colonies that showed migratory behavior were not considered for the analysis.

For observation of matrigel movements, a final concentration of 0.05% infra-red fluorescent beads (Thermo Fisher Scientific, FluoSpheres carboxylate-modified 0.2 µm, dark red 660/68) was added to matrigel prior to polymerization.

### Matrigel stiffness measurements

All matrigel samples for stiffness measurements were prepared following the same procedure as described for tissue folding settings. Then the gels were covered with PBS and measured using Chiaro Piuma nano-indenter with a probe of stiffness 0.027 N/m, tip radius 22 μm. Measurements were analyzed using DataViewerV2 (Optics11 Life).

### Optic vesicle organoids

Optic vesicle organoids were prepared following SFEBq method^41^. mES cells expressing OptoShroom3 were dissociated with 0.25% trypsin and quickly reaggregated in a differentiation medium, G-MEM supplemented with 1.5% knockout serum replacement, nonessential amino acids, sodium pyruvate, and β-mercaptoethanol (0.1 mM). 3000 cells were cultured in 100 µl media per well of a 96 well low attachment U bottom plate (Thermo). Matrigel (1%, Corning #356231) was added the next day.

Although the ES cells express the retinal marker Rx-GFP during the optic vesicle formation, the Rx-GFP expression is much lower than that of GFP-NShroom3-iLID and thus negligible. Measurements for neuroepithelial thickening and lumen reduction were manually performed using FIJI. Each measurement was performed three times and averaged.

For Supplementary movie 11, 2 µM SiR-tubulin (Spirochrome) was added to day-6 organoids and incubated for 1 h before stimulation.

### Neuroectodermal organoids

Neuroectodermal organoids were prepared using hiPS cells expressing OptoShroom3 following the first steps of cerebral organoid protocol^57^ with STEMdiff™ Cerebral Organoid Kit (STEMCELL Technologies, #08570). The modifications from the commercial protocol were: 2000 cells were used as the starting number, Y-27632 (10 µM) was added from day 0 to day 3, and the use of Accutase for cell disaggregation.

### Statistics and reproducibility

No statistical method was used to predetermine the sample size. All experiments were performed at least three times except for Supplementary movie 11 (two times). In tissue folding experiments, some MDCK colonies on matrigel presented a migratory behavior. Because this migration made them move out of the stimulation and imaging area, we decided to exclude them. The experiments were not randomized. The Investigators were not blinded to allocation during experiments and outcome assessment.

### Reporting summary

Further information on research design is available in the Nature Research Reporting Summary linked to this article.

## Supplementary information

Supplementary Information

Reporting Summary

Supplementary Movie 1

Supplementary Movie 2

Supplementary Movie 3

Supplementary Movie 4

Supplementary Movie 5

Supplementary Movie 6

Supplementary Movie 7

Supplementary Movie 8

Supplementary Movie 9

Supplementary Movie 10

Supplementary Movie 11

Supplementary Movie 12

Description of Additional Supplementary Files

## Data Availability

Source data are provided with this paper. Due to the large file size, raw image data can be obtained upon request. Please contact Miki Ebisuya or Guillermo Martínez-Ara to request data. A response will be provided in less than 2 weeks. Source data are provided with this paper.

## Source data

Source Data

## Code availability

The custom Python scripts used are available from Github [https://github.com/mebisuya/OptoShroom3], with identifier (Zenodo, 10.5281/zenodo.7031023, 2022)^58^.
