# Supplementary material for: Optogenetic control of apical constriction induces synthetic morphogenesis in mammalian tissues
Source: Nat Commun. 2022 Sep 14;13:5400. doi: 10.1038/s41467-022-33115-0 (PMC9474505; doi:10.1038/s41467-022-33115-0)
Supplement: Supplementary file 1 — Supplementary Information [file 41467_2022_33115_MOESM1_ESM.pdf]

Supplementary Information

SUPPLEMENTARY FIGURES

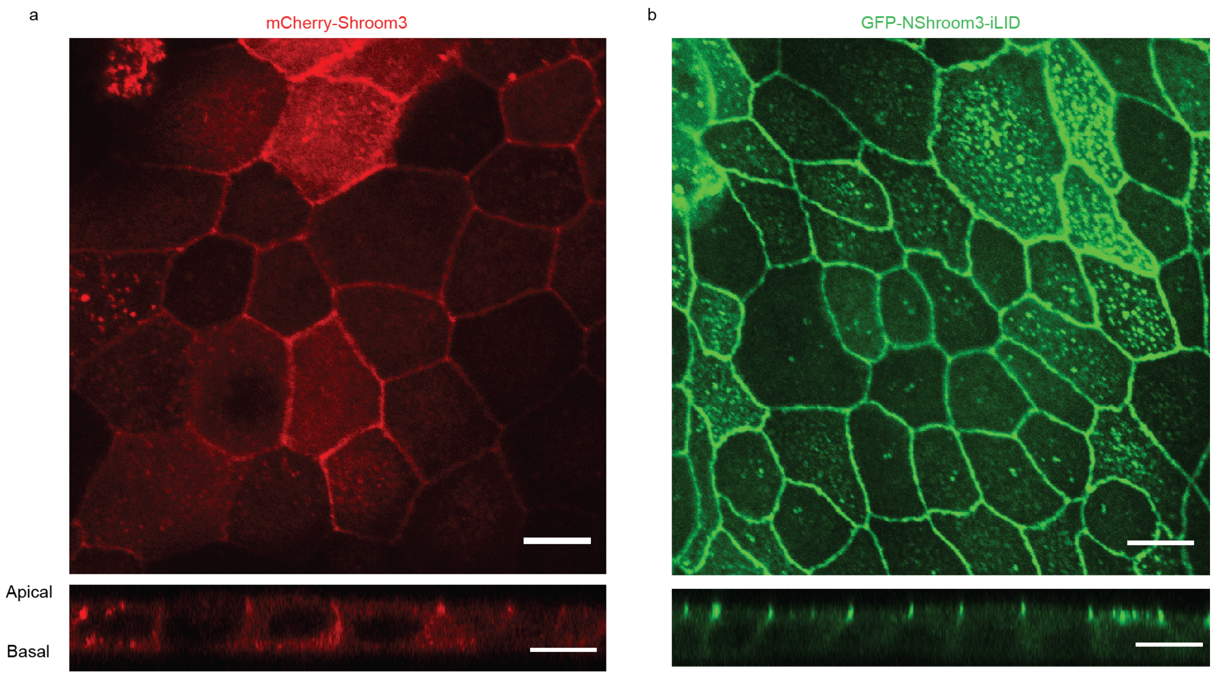

Supplementary figure 1. GFP-NShroom3-iLID localizes in the apical junctions, similarly to wild-type Shroom3. a, MDCK cells expressing mCherry-Shroom3 (wild-type). **b**, MDCK cells expressing GFP-NShroom3-iLID (same image as in figure 1c). Top: x-y apical slice, Bottom: x-z lateral slice. Scale bars = 10 µm. Representative images, both experiments were performed N >3.

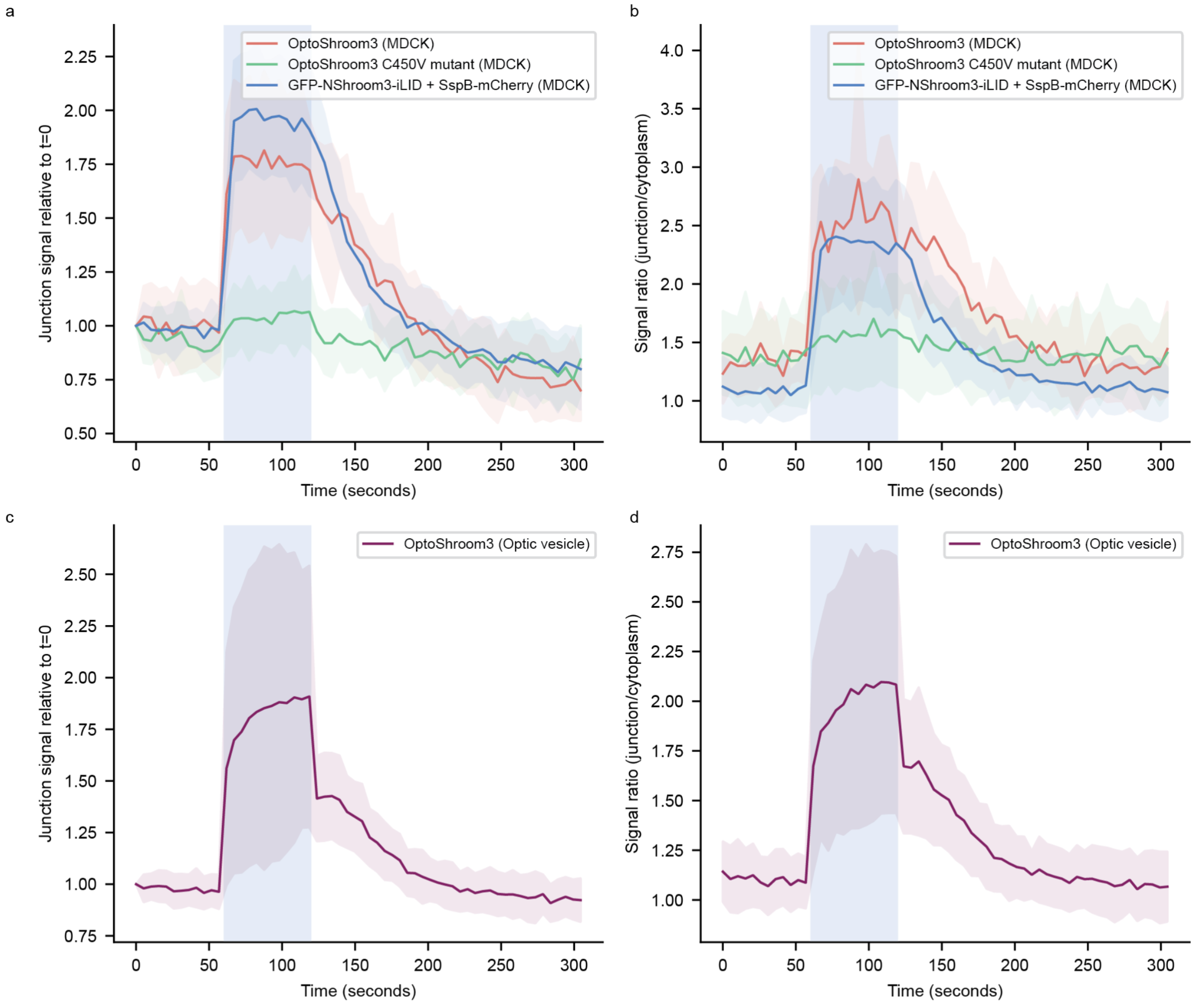

Supplementary figure 2. Translocation dynamics of SspB-mCherry-CShroom3. a, Apical junction signal of SspB-mCherry-CShroom3 relative to t = 0 upon blue light stimulation for OptoShroom3, OptoShroom3 C450V mutant, or OptoShroom3 lacking CShroom3 component in MDCK cells ($N_{OptoShroom3}$ = 6, $N_{C450Vmutant}$ = 6, $N_{SspB\text{-}mCherry}$ = 6, 3 areas measured on each sample). **b**, Junction to cytoplasmic signal ratio for the same samples as a. **c**, Apical junction signal of SspB-mCherry-CShroom3 relative to t = 0 upon blue light stimulation in optic vesicle organoids (N_{Eyecup} = 10, 3 areas measured on each sample). **d**, Junction to cytoplasmic signal ratio for optic vesicle organoids. All panels show average ± sd.

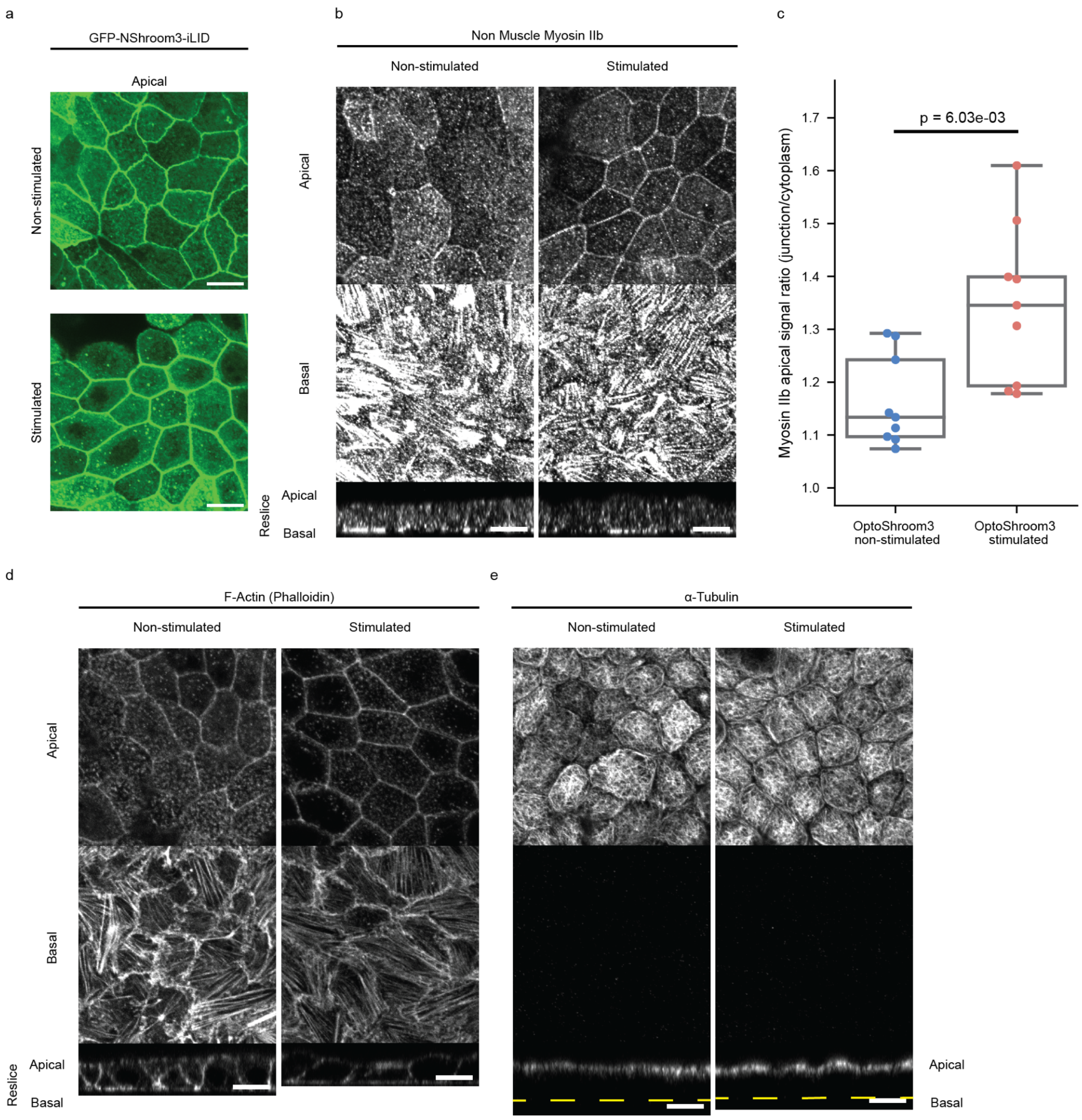

Supplementary figure 3. Effect of 2 hours of OptoShroom3 activation on cytoskeletal organization of MDCK cells. a, GFP-NShroom3-iLID signal in stimulated and non stimulated MDCK cells, projection of 3 apical slices (N = 3). **b**, Immuno-fluorescence staining of non-muscle myosin IIb in OptoShroom3 MDCK cells stimulated and non-stimulated (N = 3). **c**, Quantification of non-muscle myosin IIb apical junctions/cytoplasm signal ratio ($N_{experiments}$ = 3, 3 different areas were imaged per experiment, student's t-test). Boxplot format: the box extends from the first to the third quartile, central line shows the median. The whiskers extend from the box by 1.5x the inter-quartile range. Minima and maxima are displayed by the dotplot. **d,e**, Immuno-fluorescence staining of

F-actin, and α-tubulin in OptoShroom3 MDCK cells stimulated and non-stimulated. Yellow dashed lines mark the glass surface in panel e. Scale bars = 10 µm. (for each condition, N = 3).

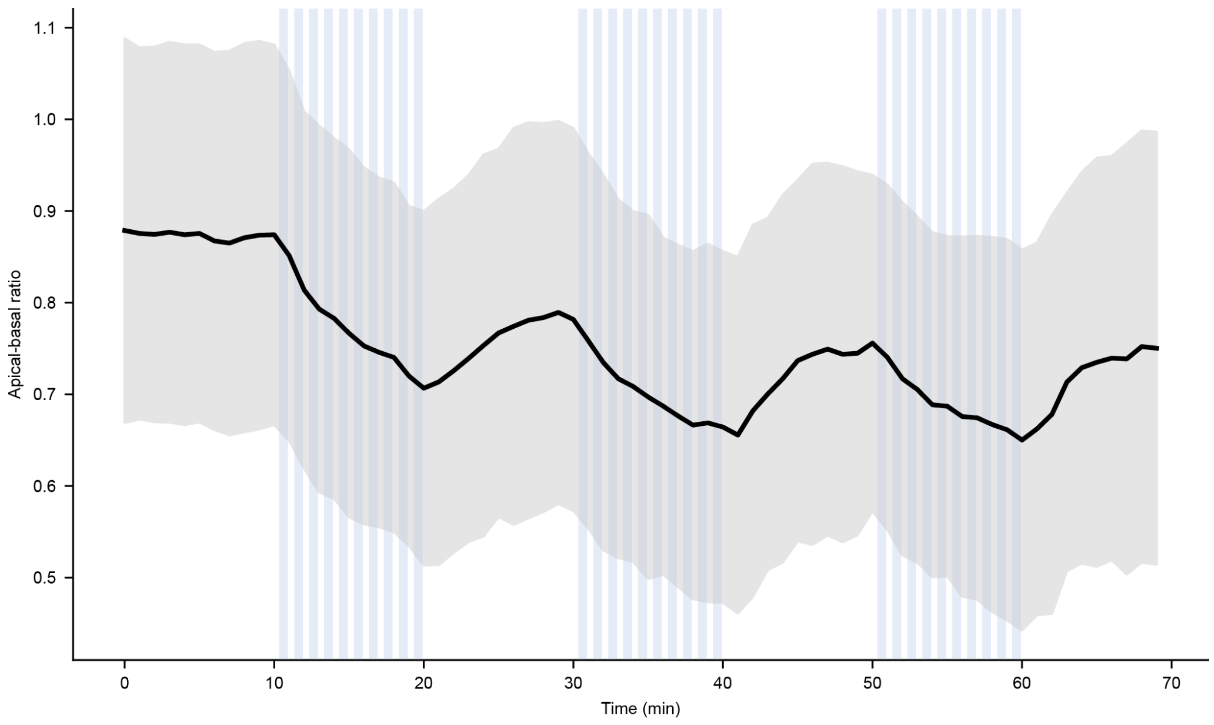

Supplementary figure 4. Apical-basal ratio reduction upon stimulation and partial recovery during non-stimulation phases. Calculated from data displayed in figure 1h, the ratio of apical and basal areas measured by segmentation of MDCK cells. Stimulation cycle: 25-second image acquisition, 35-second stimulation (blue). Repetition of 10 cycles of rest (no stimulation) and 10 cycles of stimulation (N = 8, avg ± sd).

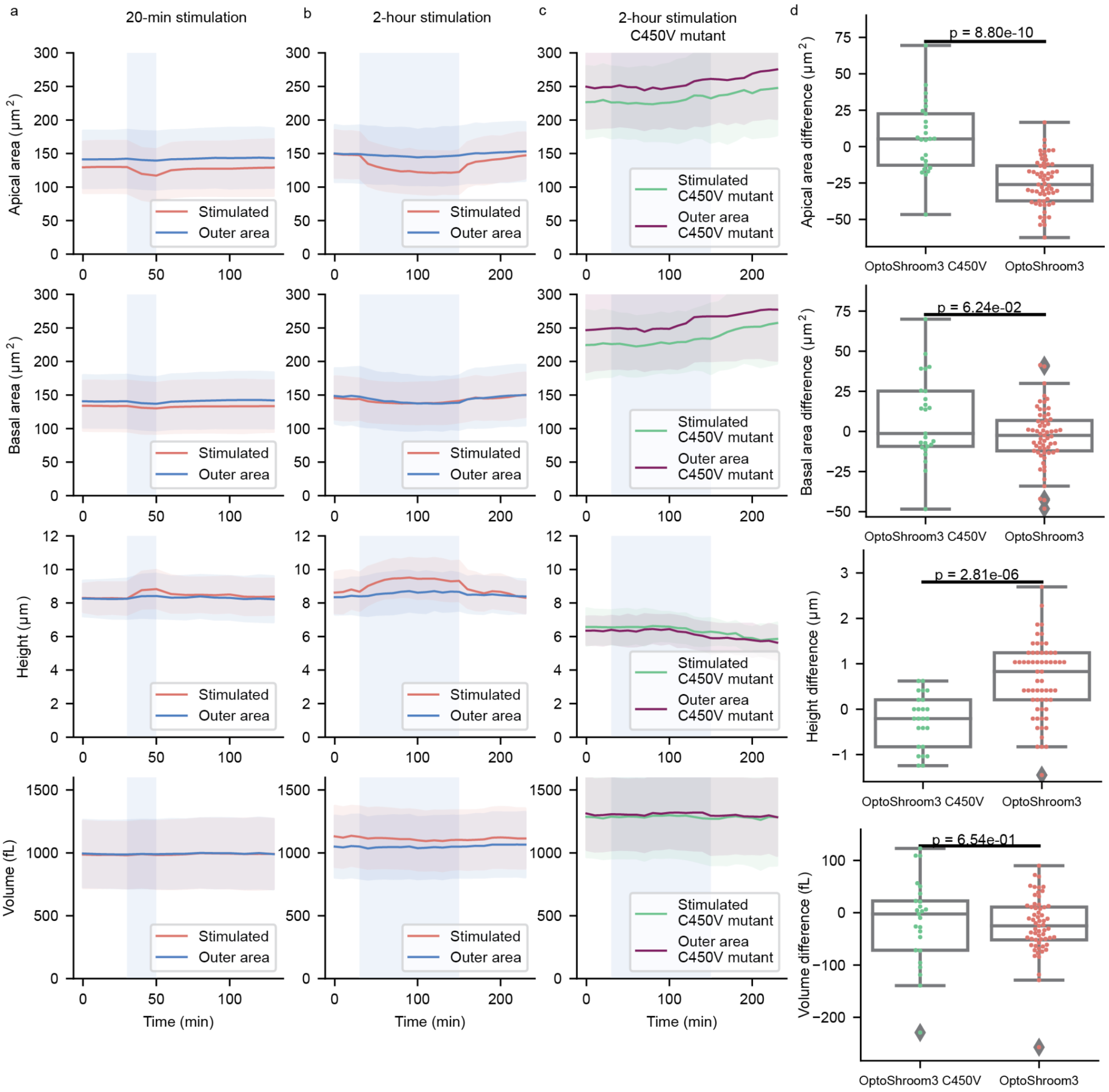

Supplementary figure 5. Quantification of apical and basal area, height, and volume in MDCK cells. **a**, 20-minute stimulation of OptoShroom3 MDCK cells ($N_{Experiments}$ = 6, $N_{Stimulated}$ = 74, N_{Outer} =202, avg ± sd). **b**, 2-hour stimulation of OptoShroom3 cells ($N_{Experiments}$ = 5, $N_{Stimulated}$ = 61, N_{Outer} =137, avg ± sd). This panel is also displayed in figure 2. **c**, 2-hour stimulation of OptoShroom3 C450V mutant cells ($N_{Experiments}$ = 5, $N_{Stimulated}$ = 25, N_{Outer} = 46, avg ± sd). **d**, Comparison of OptoShroom3 and C450V mutant stimulated cells in apical and basal area, cell height, and volume. Values are differences between start and end of 2-hour stimulation ($N_{Stimulated}$ = 61, $N_{Stimulated\ C450V\ mutant}$ = 25, student's t-test, two sided). Boxplot format: the box extends from the first to the third quartile,

central line shows the median. The whiskers extend from the box by 1.5x the inter-quartile range. Minima and maxima are displayed by the dotplot.

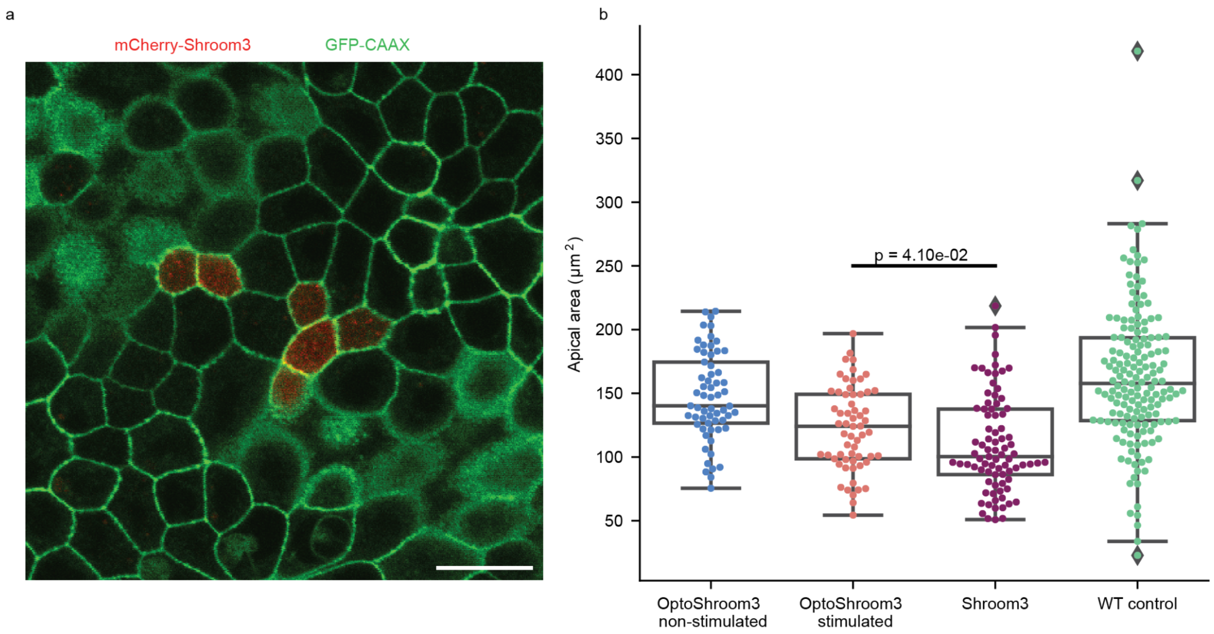

Supplementary figure 6. Comparison of apical area reduction provoked by Shroom3 and OptoShroom3. a, Representative image of sparse expression of mCherry-Shroom3 on GFP-CAAX labeled MDCK cells. Image acquired 24 hours after induction of Shroom3 expression. Scale bar = 20 µm. **b**, Comparison of apical area in MDCK cells for OptoShroom3 stimulated and non-stimulated, 24 hour induction of Shroom3 expression, and WT cells ($N_{Stimulated}$ = 61, $N_{non\text{-}timulated}$ = 61, $N_{Shroom3}$ = 83, $N_{WT\ control}$ = 161, student's t-test, two sided). OptoShroom3 stimulated and non-stimulated data belong to timepoints between start and end of 2-hour stimulation (data shown in supp. figure 5b). Boxplot format: the box extends from the first to the third quartile, central line shows the median. The whiskers extend from the box by 1.5x the inter-quartile range. Minima and maxima are displayed by the dotplot.

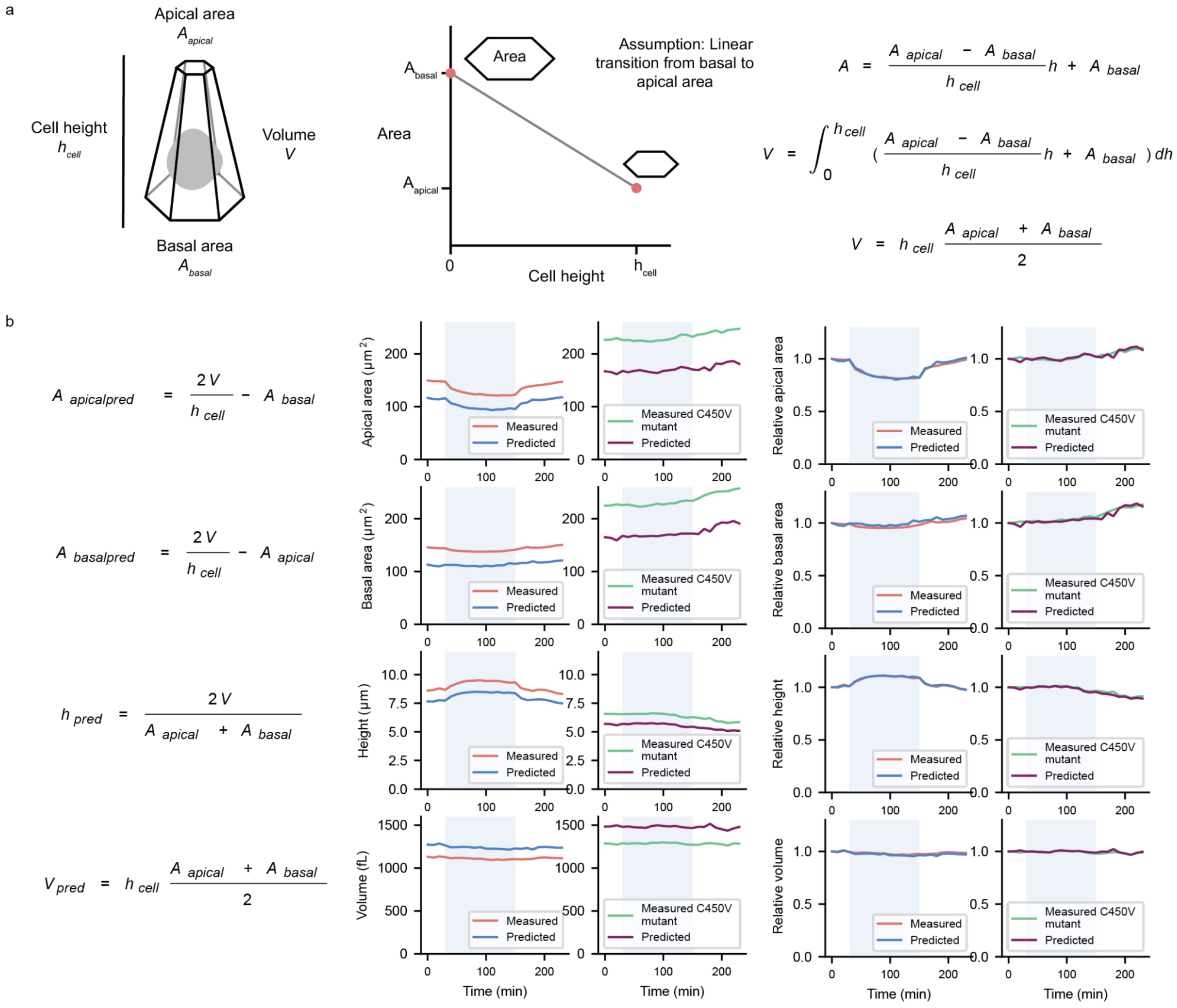

Supplementary figure 7. 3D parameter prediction matches with measurements. a, Simple model for prediction of one of the four parameters (volume, height, apical, or basal area) from the input of the other 3 measured parameters. This model is based on the assumption that the cross-sectional area linearly changes from apical to basal area. **b**, Predicted total and relative parameters for 2-hour stimulated OptoShroom3 and OptoShroom3 C450V mutant cells using mean values measured in supp. figure 5. The panel for absolute height is also displayed in figure 2.

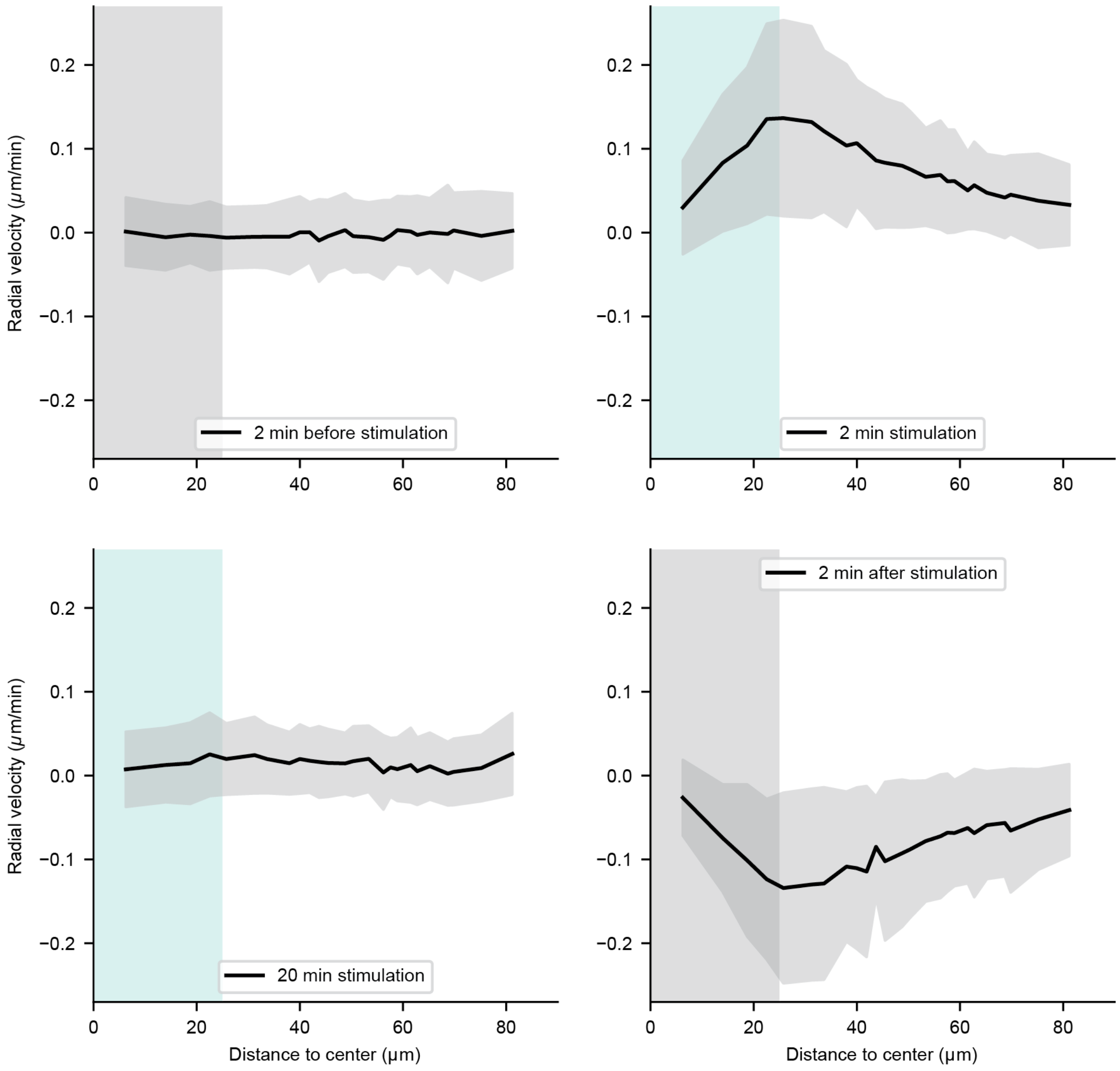

Supplementary figure 8. OptoShroom3 induced apical displacement averaged by distance to the center of stimulation. Average displacements caused by stimulation of a 35.3 × 35.3 µm square on confluent MDCK cells on 100% collagen gel before stimulation, after 2 minutes of stimulation, after 20 minutes of stimulation, and 2 minutes after the end of stimulation. Stimulated area is displayed in blue as the distance from the center to the corner of the square (24.96 µm) (N = 6, avg ± sd) (Reanalysis of data from figure 3).

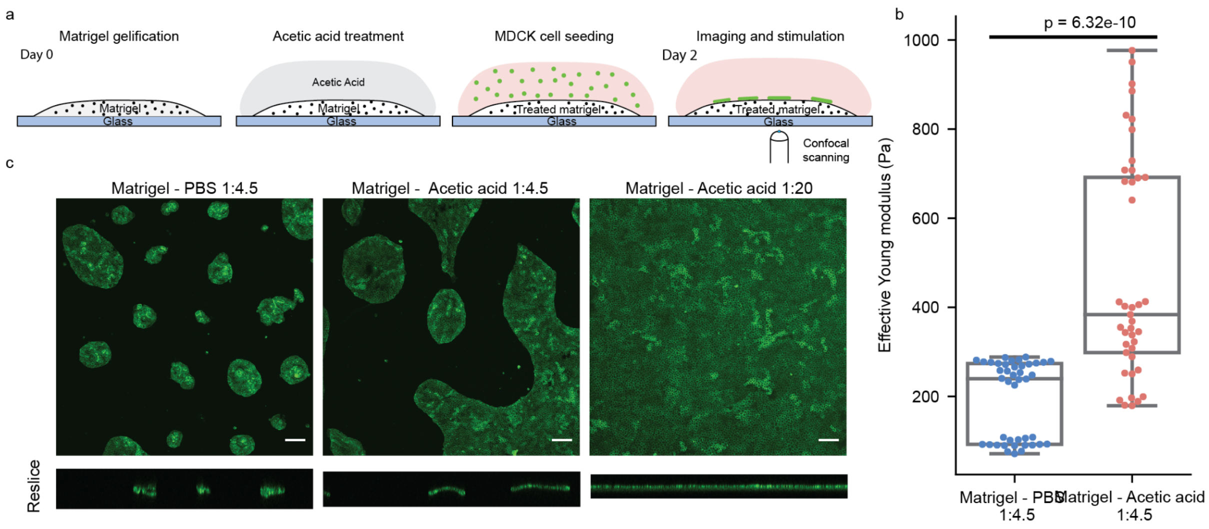

Supplementary figure 9. Acetic acid treatment of matrigel affects MDCK colony formation. a, Protocol to improve flat MDCK monolayer formation on matrigel settings. A thick matrigel gel was polymerized on a glass-bottom dish, then incubated with 20 mM acetic acid before cell seeding. **b**, Quantification of the impact of acetic acid treatment 20 mM, 4.5x acetic acid/matrigel volume ratio on gel stiffness (N_{PBS} = 4, $N_{Acetic\ acid}$ = 4, each sample measured at least 7 times on different areas, student's t-test, two sided). Boxplot format: the box extends from the first to the third quartile, central line shows the median. The whiskers extend from the box by 1.5x the inter-quartile range. Minima and maxima are displayed by the dotplot. **c**, Comparison of MDCK colonies formed on untreated (PBS) matrigel with those formed on gels treated with two different volumes of acetic acid (acetic acid/matrigel = 4.5 and 20). Same number of cells was seeded on the three gels. GFP-CAAX signal. Scale bar = 100 µm.

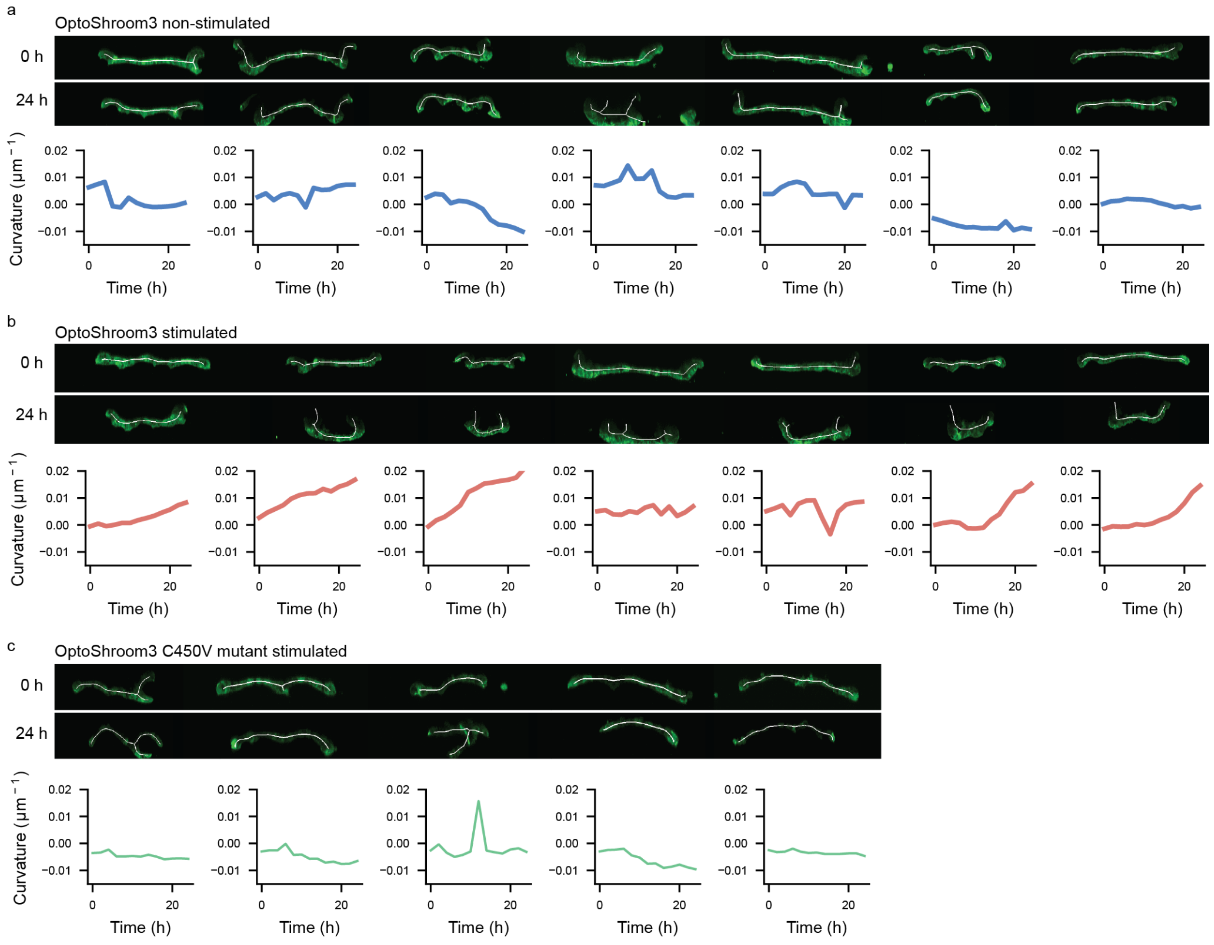

Supplementary figure 10. Summary of MDCK cell colony folding. a-c, Summary of average curvature measurements from the centric line of non-stimulated, stimulated and stimulated C450V mutant colonies averaged in figure 4 measurements ($N_{\text{Non-stimulated}}$ = 7, $N_{\text{Stimulated}}$ = 7, $N_{\text{C450Vmutant}}$ = 5) (Raw data from figure 4. Non-stimulated 7, stimulated 7, and stimulated C450V-mutant 2 are also displayed on figure 4).

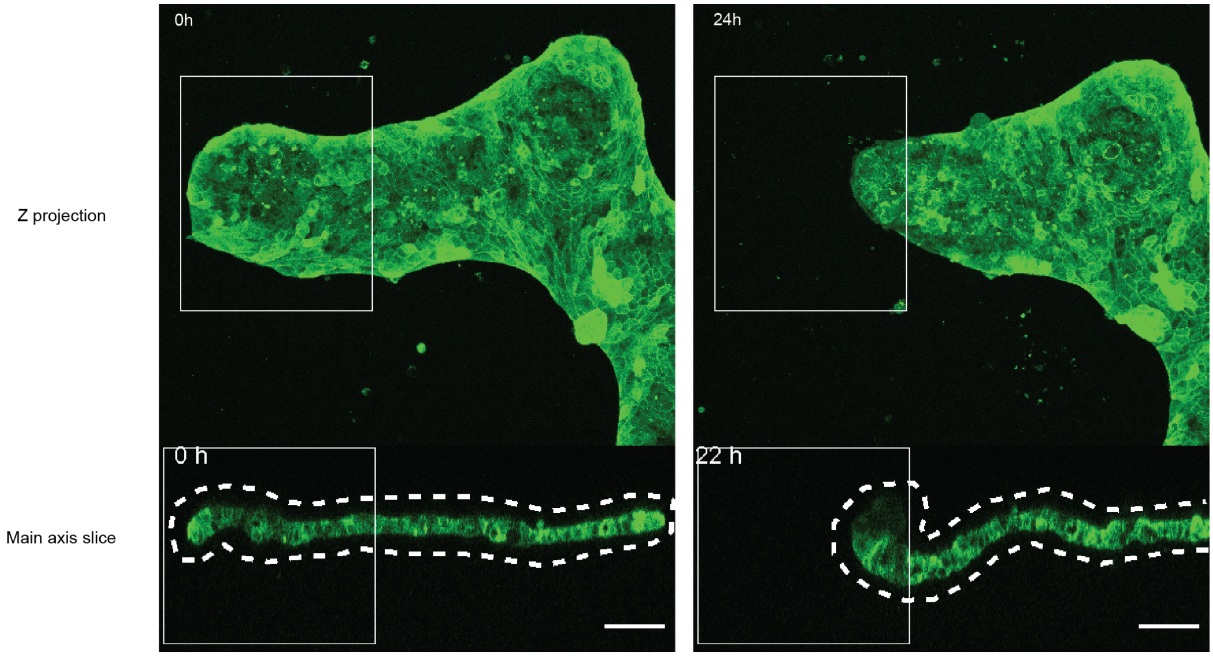

Supplementary figure 11. Induction of selective folding on MDCK monolayers. Stimulation of restricted areas of OptoShroom3 MDCK colonies on matrigel induced selective folding of the stimulated area. Scale bar = 50 µm. N > 3.

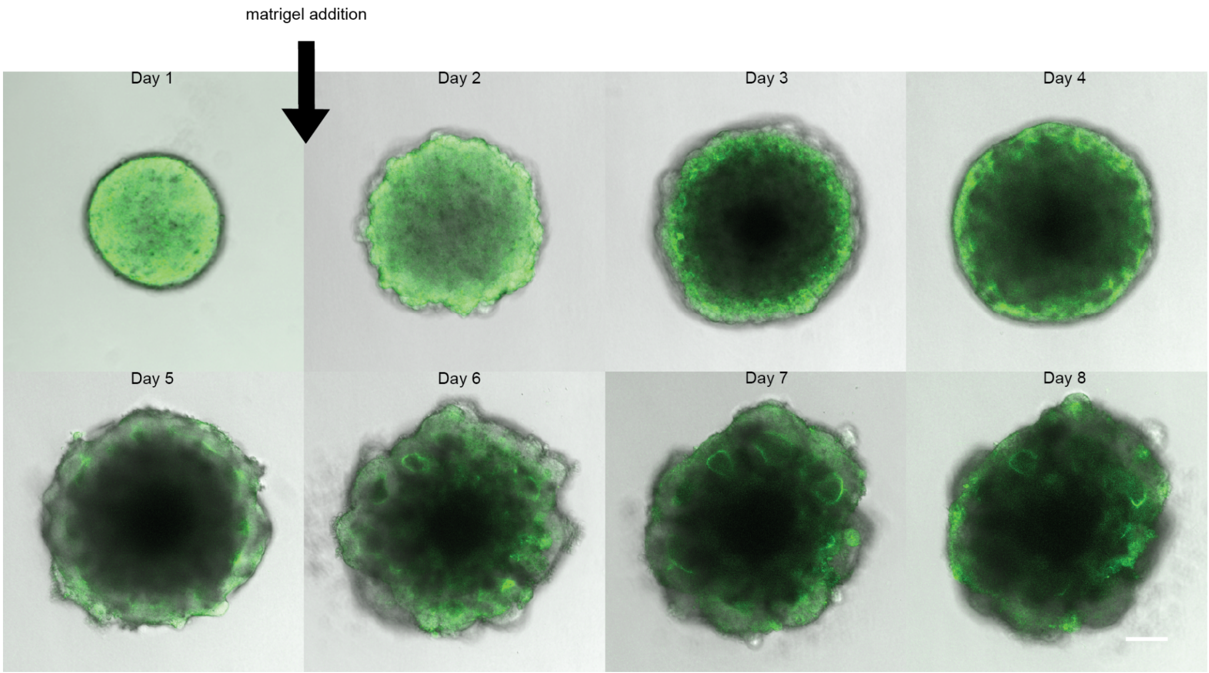

Supplementary figure 12. Optic vesicle organoid development and change in GFP-NShroom3-iLID localization. During the first 8 days of optic vesicle organoid formation, GFP-NShroom3-iLID localization changed from homogeneous distribution to a strong localization on the apical side of vesicles. Scale bar = 100 µm.

Supplementary Table 1. OptoShroom3 sequences.

Name	Vector	Sequence	Comments
GFP-NShroom3-iLID	PiggyBac, CAG promoter	atggtgagcaagggcgaggagctgttcaccggggtggtgcccatcctggtcgagctggacggcgacgtaaacggccacaagttcagcgtgtccggcgagggcgagggcgatgccacctacggcaagctgaccctgaagttcatctgcaccaccggca<br>agctgcccgtgccctggcccaccctcgtgaccaccctgacctacggcgtgcagtgcttcagccgctaccccgaccacatgaagcagcacgacttcttcaagtccgccatgcccgaaggctacgtccaggagcgcaccatcttcttcaaggacgacggca<br>actacaagacccgcgccgaggtgaagttcgagggcgacaccctggtgaaccgcatcgagctgaagggcatcgacttcaaggaggacggcaacatcctggggcacaagctggagtacaactacaacagccacaacgtctatatcatggccgacaagc<br>agaagaacggcatcaaggtgaacttcaagatccgccacaacatcgaggacggcagcgtgcagctcgccgaccactaccagcagaacacccccatcggcgacggccccgtgctgctgcccgacaaccactacctgagcacccagtccgccctgagca<br>aagaccccaacgagaagcgcgatcacatggtcctgctggagttcgtgaccgccgccgggatcactctcggcatggacgagctgtacaagggtagtggtagtggtaccatcgatatgcagatatctcagggcacaatgggccctccttggcaccagagc<br>taccattccagctcttcgaccagtgacctctccaactacgaccatgcttatctgaggcggagccctgaccagtgcagctcccaggggagcatggaaagcctggagcccagtggcggatacccaccctgccatctcctttctcctgccaagtccacgagca<br>gcatcgaccagctaggccaccttcataacaaaagggactccgcatatagctccttctccaccagttcgagcatcttcgagtacccacccccctggtggctctgcccgagaacgttcgggttccatggacgtgatttctgcccggggcggcctcctagaaggg<br>atgaggcaggcagacatccgctacgtcaagacggtctatgatacccggagaggagtatcttcagagtatgaggtgaacccttcagccttgctccttcaaggtagggatgcccatgcctcagccgacagtcagggctgtgctaaatggcacagcattcct<br>cggggcaagggaacgccttccccgtcttggagccagcagtgttctggttccctggagactgctactgacaacctgccccagaaggcaggcgcacccctgccccccacccgcagtgatagctatgcagccttccgccatcgcgagcgtcccagctcctggt<br>ccagccttgatcagaaaaggttctgccggcctcagacaaactcttcaggctcccagaaaaccccattcgctgaggaccagctgcatacagtcccagagaggagtccagagaacagcccccagtgaaatctaagcataattatacccagaaggcgca<br>gcctggccaacctctgctgcccactggcatctatccggtaccttctccggagcctcactttgcccaggtgccccagccctctgtgagtagtaatggcaccgtctaccctgcgctagtcaaagagagtggatacacagctgctcagggaacctgtaacaaga<br>tggctaccttagatgagaacgggaatcaaaatgaagctagcaggcctgggtttgccttctgtcagcccctagaacacaactcggtgactccggtggagaaaaggccagaacccacggccaaatacatctacaaagtccatttttcttcagtgcctgaaa<br>atgaagactcctccctaaagaggcacatcacacctccccacggccatagtccatatcccagcgagagaaagaacatccacggtggcagcagagcgtgttccaatcaccacagtctctcgtcccccaggcccaggccctgcacgtgggtgatgacagg<br>aggccttccaggctctctcagccctgggagggtgactttcaggaagatcacaatgccaacctcaggcagaaagtggagagggagggccaaggccagggcctgtcgggcaactctggcaggaccagatcggccttctcatctctgcagaatattccaga<br>gagtctgagaagacaaagcaacgtggagcttggagaagcccaggaggttcaccccgggggcaggtccaaggtggaggaccctggaaggaaagcaggggcttcagacatcaggggctacctggacagatctgtttcctatccgaggcccgaggga<br>aaatgaacgccgtagactccgtccacagtgcagactccaggtatgaggagtccccggccccagcgctgccacagacatccggtgcgagccagcggcggctcagctccagcagctccgccgccccgcagtacaggaagccccattgctccgtgctgga<br>gaaggtttctagaatagaggaacgagagcaggggcgccacagacccctgagcgtgggcagctctgcatacggccccggctacaggcctgggaggaccggcccgacccccagtacttccagcagtgacctcgacgatccaaaagccggcagcgtcca<br>tttctcggagtccactgaacacctgcgcaatggggagcagaacccccaaacggggaggctaagcaggaagaggcttcccggccccagtgcagccatttgatccggagggcccctgcggacggccgtggacctcctgcccgaggcggcgagccctca<br>aggccggaggcccgcctgctccgcagccagagcacctttcagctctacagtgaggcggagagggaagcctcgtggtcagaggacaggcccggtacccctgagtcgcccctgctcgatgcgcctttcagccgcgcctacagaaacagcatcaaggatgc<br>ccagtcccgcgtcctgggggccacgtcgtttcgtcgccgagacctggaaccgggcacccccgccacctcgaggccctggcgcccgcgacccgcctccgcccacgtggggatgcggagccccgaggccgcggtgccctcgtcctccccgcacacccctcg<br>ggagcgacacagcgtgaccccggccgcgccccaggctgcccgccgaggcccacgccggcgcctcactgtcgagcagaagaagcgctcctactcggagcccgagaagatgaacgaggtaggggtctcggaggaggccgagcccacgccctgcggcc<br>cgccgaggcccgcgcagccgcgcttctcggagagcaccgtggccgaccggcgccgcatcttcgagcgcgacggcaaggcctgctccacgctcagcctgtcgggccccgagctcaagcagttccagcagagcgccctggccgactacatccagcgtaa<br>gacgggcaagcggcccacaggagccgcctgcacaccggaggccgggctgcgcgagcgcgcacagagcgcctacctccaggccggccccgcggcgcccgatggccccggcctggcctctgcctgtagcctgagctccctgcgagagcccgaggcgct<br>gccccgcaaggagcacactcatccgtcggccgcggacggcccgcaggcgccccgtgatcgcagcagctccttcgccagcggacgcctagtcggggagcgacgtcgctgggaccctcaggtccccaggcagctgctcagcggagcgaactgcgaaccg<br>aggggcgtccagaggatggacggggccccgggcgggccgccttcctggggcatggtggccgggaaggccgggaagtccaagtcagccgaggatctgctggagcgctccgacacactggcggtccctgtgcatgtgaggtccaggtcgtctcccacct<br>ccgacaagaaaggtcaggatgtgctcctgagggaaggcagcaactttggttttgtaaaggatccatgctgtttggctggccctggacctaggtctctcagctgctcagacaaaggccagaatgagctggcattgcccctgcaccaccctacgccctgctg<br>gaacggctcaggctgcaaagccactgtggcttcctcagctcctcccgaatcttctggagccgcggatcatctgaagcaacgtagagcgcctggtcctaggccactctcagcaggaatgcacggccacttcccagacgccagagctgcctcactcagctcc<br>cctctgccatcacctgtcccctccagttaccggtcacagcttgccatggatcagcagactgggcaacagcctccctcatcccctgcctctgcagtgacccagcctaccagtccaagaagccctgagctcagcagcccagcatatgggctagagaaggga<br>tgtggaagaggacatccctgcctcagcaacccccctcccctttgggtgaaatgggcccacgcggtcagagaagacggccttgctgaggacaccttggcgcctgagtttgcgaacctgaagcactatagaaaccagccgagtcttccgagttcctgcagca<br>cttctgacccagacactccaggaaggatctcgctccgaatatcggagtcagcccttcaaccttccccaccgccccggggggactatgatgacgaagtgttcatgaaggacttgcaccccaaggttacttcaagccccacatttgaagctcttcccccaccc<br>ccgccaccttcgccaccgagcgaggaacctctagtgaatggcacagatgactttcctccgcctcctcctccccaagccctgtgtgaggtactgctggatggtgaggcatccacggaagctggcagcggcccctgcagaattcccagggtgatggtcacat<br>ccggaggggatcggtggagctccaccggtggcggccgctctagagagaatctttattttcagggcgccgccaaattcaaagaaaccgctgctgctaaattcgaacgccagcacatggacagcggaggtggaggtactagtctcgagggtagtggtagt<br>ggggagtttctggcaaccacactggaacggatcgagaaaaatttcgtgattactgatccgagactgcctgacaacccaatcatttttgcgagcgattccttcctgcagctgacagaatattctcgggaagagatcctggggcgcaattgccgttttctgca<br>gggacccgagacagaccgtgccactgttcggaaaatcagagatgctattgacaaccagactgaagtgaccgttcagctgatcaattataccaagagcggcaagaagttctggaacgtgttccacctgcagccgatgcgcgattataagggcgacgtcc<br>agtacttcattggcgtgcagctggatggcaccgaacgtcttcatggcgccgctgagcgtgaggcggtctgcctgatcaaaaagacagcctttcagattgctgaggcagcgaacgacgaaaattacttttga	Combination of sequences: eGFP, mSHROOM3 until T1387, and iLID.
SspB-mCherry-CShroom3	PiggyBac, CAG promoter	atggccaccgggagctccccgaaacgccctaagctgctgcgtgaatattacgattggctggttgataacagctttaccccatatctggtggtggatgccacatacctgggcgtgaacgtgcccgtggagtatgtgaaagacggtcagatcgtgctgaatc<br>tgtctgcaagtgcgaccggcaacctgcaactgacaaatgattttatccagttcaacgcccgctttaagggcgtgtctcgtgaactgtatatcccgatgggtgccgctctggccatttacgctcgcgagaacggcgatggtgtgatgttcgaaccagaagaa<br>atctatgacgagctgaatattggtcccggtgaattcggagggggcagtggtggtggatctatggtgagcaagggcgaggaggataacatggccatcatcaaggagttcatgcgcttcaaggtgcacatggagggctccgtgaacggccacgagttcga<br>gatcgagggcgagggcgagggccgcccctacgagggcacccagaccgccaagctgaaggtgaccaagggtggccccctgcccttcgcctgggacatcctgtcccctcagttcatgtacggctccaaggcctacgtgaagcaccccgccgacatccccg<br>actacttgaagctgtccttccccgagggcttcaagtgggagcgcgtgatgaacttcgaggacggcggcgtggtgaccgtgacccaggactcctccctgcaggacggcgagttcatctacaaggtgaagctgcgcggcaccaacttcccctccgacggcc<br>ccgtaatgcagaagaagaccatgggctgggaggcctcctccgagcggatgtaccccgaggacggcgccctgaagggcgagatcaagcagaggctgaagctgaaggacggcggccactacgacgctgaggtcaagaccacctacaaggccaagaa<br>gcccgtgcagctgcccggcgcctacaacgtcaacatcaagttggacatcacctcccacaacgaggactacaccatcgtggaacagtacgaacgcgccgagggccgccactccaccggcggcatggacgagctgtacaagggatccgatcccgggggg<br>agggggcagtggtggtggatctatgagggaagggcacgtgcctggggcagcccattccgaaggtagtcagataatgactgccacaccaccccaaacctcggccaaaggttcagaagccgagtccaacacgccttccagcgcgagcgcccagcctcag<br>ctcaacggttctcctggcaagcagctctgtcccagccagacccgaaacctcacttatgaacctgtcgagagaactcaggatctaggaaagaaaactcacgccgagcctcagaagacctcagaagatatccggacagaggctctagccaaggaaattgt<br>ccaccaagacaagtctctggcagacattttagatcctgactccaggatgaagacaacgatggacctgatggagggtttattccctggagacgccagtgtgctgatggatagtggcgcaaagaggaaagccttagacatcaccgccaggcgtgcgggat<br>gtgaagccaaggcgagcgaccacaaggaggcagtgagtgtgctggtcaactgtcctgcctactacagtgtctctgctgccaaggcggagctgctcaacaaaatcaaagacatgccggaggagctgcaggaggaggagggcaggaggatgtcaatg<br>agaaaaaggctgagcttatcggaagcctcacccacaagctggagagtctccaggaagccaaggggagcctgctcacggacatcaagctgaacaatgccctgggagaggaggtggaggctctgatcagtgagctctgcaaacccaacgagtttgaca<br>agtacaagatgttcataggggacctggacaaggtggtcaacctgctgctgtccctgtctggacgcctggcccgcgtggagaacgtcctcagaggccttggtgaagacgccagcaaagaggaaaggagttctctgaacgagaaaaggaaggttctggct<br>ggccagcacgaggacgcacgggagctcaaggagaacctggaccggagggagcgcgtggtgctggatatcctggccaactacctgtccgccgagcagctgcaggactaccagcacttcgtgaagatgaagtccacactcctcattgagcagcggaag<br>ctggatgacaagataaagctgggccaggagcaggtcaggtgcctgctggaatcgcttccctcggacttcaggcccaaggcaggggccatctccctccctccagccctcaccggccatgcgactcctgggggaacatctgtattcggtggcgttttcccaa<br>cgttgacctctcctcttcccgggtaa	Combination of sequences: SspB, mCherry and mSHROOM3 from R1388.
GFP-NShroom3-iLID, C450V mutant	PiggyBac, CAG promoter	actagtatggtgagcaagggcgaggagctgttcaccggggtggtgcccatcctggtcgagctggacggcgacgtaaacggccacaagttcagcgtgtccggcgagggcgagggcgatgccacctacggcaagctgaccctgaagttcatctgcacca<br>ccggcaagctgcccgtgccctggcccaccctcgtgaccaccctgacctacggcgtgcagtgcttcagccgctaccccgaccacatgaagcagcacgacttcttcaagtccgccatgcccgaaggctacgtccaggagcgcaccatcttcttcaaggacg<br>acggcaactacaagacccgcgccgaggtgaagttcgagggcgacaccctggtgaaccgcatcgagctgaagggcatcgacttcaaggaggacggcaacatcctggggcacaagctggagtacaactacaacagccacaacgtctatatcatggccg<br>acaagcagaagaacggcatcaaggtgaacttcaagatccgccacaacatcgaggacggcagcgtgcagctcgccgaccactaccagcagaacacccccatcggcgacggccccgtgctgctgcccgacaaccactacctgagcacccagtccgccc<br>tgagcaaagaccccaacgagaagcgcgatcacatggtcctgctggagttcgtgaccgccgccgggatcactctcggcatggacgagctgtacaagggtagtggtagtggtaccatcgatatgcagatatctcagggcacaatgggccctccttggcac<br>cagagctaccattccagctcttcgaccagtgacctctccaactacgaccatgcttatctgaggcggagccctgaccagtgcagctcccaggggagcatggaaagcctggagcccagtggcggatacccaccctgccatctcctttctcctgccaagtcca<br>cgagcagcatcgaccagctaggccaccttcataacaaaagggactccgcatatagctccttctccaccagttcgagcatcttcgagtacccacccccctggtggctctgcccgagaacgttcgggttccatggacgtgatttctgcccggggcggcctccta<br>gaagggatgaggcaggcagacatccgctacgtcaagacggtctatgatacccggagaggagtatcttcagagtatgaggtgaacccttcagccttgctccttcaaggtagggatgcccatgcctcagccgacagtcagggctgtgctaaatggcacag<br>cattcctcggggcaagggaacgccttccccgtcttggagccagcagtgttctggttccctggagactgctactgacaacctgccccagaaggcaggcgcacccctgccccccacccgcagtgatagctatgcagccttccgccatcgcgagcgtcccagc<br>tcctggtccagccttgatcagaaaaggttctgccggcctcagacaaactcttcaggctcccagaaaaccccattcgctgaggaccagctgcatacagtcccagagaggagtccagagaacagcccccagtgaaatctaagcataattatacccagaa<br>ggcgcagcctggccaacctctgctgcccactggcatctatccggtaccttctccggagcctcactttgcccaggtgccccagccctctgtgagtagtaatggcaccgtctaccctgcgctagtcaaagagagtggatacacagctgctcagggaacctgta<br>acaagatggctaccttagatgagaacgggaatcaaaatgaagctagcaggcctgggtttgccttctgtcagcccctagaacacaactcggtgactccggtggagaaaaggccagaacccacggccaaatacatctacaaagtccatttttcttcagtgc<br>ctgaaaatgaagactcctccctaaagaggcacatcacacctccccacggccatagtccatatcccagcgagagaaagaacatccacggtggcagcagagcgtgttccaatcaccacagtctctcgtcccccaggcccaggccctgcacgtgggtgat<br>gacaggaggccttccaggctctctcagccctgggagggtgactttcaggaagatcacaatgccaacctcaggcagaaagtggagagggagggccaaggccagggcctgtcgggcaactctggcaggaccagatcggccttctcatctctgcagaata<br>ttccagagagtctgagaagacaaagcaacgtggagcttggagaagcccaggaggttcaccccgggggcaggtccaaggtggaggaccctggaaggaaagcaggggcttcagacatcaggggctacctggacagatctgtttcctatccgaggcccg<br>aggggaaaatgaacgccgtagactccgtccacagtgcagactccaggtatgaggagtccccggccccagcgctgccacagacatccggtgcgagccagcggcggctcagctccagcagctccgccgccccgcagtacaggaagccccattgctccgt<br>gctggagaaggtttctagaatagaggaacgagagcaggggcgccacagacccctgagcgtgggcagctctgcatacggccccggctacaggcctgggaggaccggcccgacccccagtacttccagcagtgacctcgacgatccaaaagccggcag<br>cgtccatttctcggagtccactgaacacctgcgcaatggggagcagaacccccaaacggggaggctaagcaggaagaggcttcccggccccagtgcagccatttgatccggagggcccctgcggacggccgtggacctcctgcccgaggcggcgag<br>ccctcaaggccggaggcccgcctgctccgcagccagagcacctttcagctctacagtgaggcggagagggaagcctcgtggtcagaggacaggcccggtacccctgagtcgcccctgctcgatgcgcctttcagccgcgcctacagaaacagcatcaa<br>ggatgcccagtcccgcgtcctgggggccacgtcgtttcgtcgccgagacctggaaccgggcacccccgccacctcgaggccctggcgcccgcgacccgcctccgcccacgtggggatgcggagccccgaggccgcggtgccctcgtcctccccgcaca<br>cccctcgggagcgacacagcgtgaccccggccgcgccccaggctgcccgccgaggcccacgccggcgcctcactgtcgagcagaagaagcgctcctactcggagcccgagaagatgaacgaggtaggggtctcggaggaggccgagcccacgccct<br>gcggcccgccgaggcccgcgcagccgcgcttctcggagagcaccgtggccgaccggcgccgcatcttcgagcgcgacggcaaggcctgctccacgctcagcctgtcgggccccgagctcaagcagttccagcagagcgccctggccgactacatcca<br>gcgtaagacgggcaagcggcccacaggagccgcctgcacaccggaggccgggctgcgcgagcgcgcacagagcgcctacctccaggccggccccgcggcgcccgatggccccggcctggcctctgcctgtagcctgagctccctgcgagagcccga<br>ggcgctgccccgcaaggagcacactcatccgtcggccgcggacggcccgcaggcgccccgtgatcgcagcagctccttcgccagcggacgcctagtcggggagcgacgtcgctgggaccctcaggtccccaggcagctgctcagcggagcgaactgc<br>gaaccgaggggcgtccagaggatggacggggccccgggcgggccgccttcctggggcatggtggccgggaaggccgggaagtccaagtcagccgaggatctgctggagcgctccgacacactggcggtccctgtgcatgtgaggtccaggtcgtctc<br>ccacctccgacaagaaaggtcaggatgtgctcctgagggaaggcagcaactttggttttgtaaaggatccatgctgtttggctggccctggacctaggtctctcagctgctcagacaaaggccagaatgagctggcattgcccctgcaccaccctacgcc<br>ctgctggaacggctcaggctgcaaagccactgtggcttcctcagctcctcccgaatcttctggagccgcggatcatctgaagcaacgtagagcgcctggtcctaggccactctcagcaggaatgcacggccacttcccagacgccagagctgcctcactc<br>agctcccctctgccatcacctgtcccctccagttaccggtcacagcttgccatggatcagcagactgggcaacagcctccctcatcccctgcctctgcagtgacccagcctaccagtccaagaagccctgagctcagcagcccagcatatgggctagaga<br>aggggatgtggaagaggacatccctgcctcagcaacccccctcccctttgggtgaaatgggcccacgcggtcagagaagacggccttgctgaggacaccttggcgcctgagtttgcgaacctgaagcactatagaaaccagccgagtcttccgagttcct<br>gcagcacttctgacccagacactccaggaaggatctcgctccgaatatcggagtcagcccttcaaccttccccaccgccccggggggactatgatgacgaagtgttcatgaaggacttgcaccccaaggttacttcaagccccacatttgaagctcttcc<br>cccacccccgccaccttcgccaccgagcgaggaacctctagtgaatggcacagatgactttcctccgcctcctcctccccaagccctgtgtgaggtactgctggatggtgaggcatccacggaagctggcagcggcccctgcagaattcccagggtgatg<br>gtcacatccggaggggatcggtggagctccaccggtggcggccgctctagagagaatctttattttcagggcgccgccaaattcaaagaaaccgctgctgctaaattcgaacgccagcacatggacagcggaggtggaggtactagtctcgagggtag<br>tggtagtggggagtttctggcaaccacactggaacggatcgagaaaaatttcgtgattactgatccgagactgcctgacaacccaatcatttttgcgagcgattccttcctgcagctgacagaatattctcgggaagagatcctggggcgcaatgtgcgtt<br>ttctgcagggacccgagacagaccgtgccactgttcggaaaatcagagatgctattgacaaccagactgaagtgaccgttcagctgatcaattataccaagagcggcaagaagttctggaacgtgttccacctgcagccgatgcgcgattataagggc<br>gacgtccagtacttcattggcgtgcagctggatggcaccgaacgtcttcatggcgccgctgagcgtgaggcggtctgcctgatcaaaaagacagcctttcagattgctgaggcagcgaacgacgaaaattacttttga	Combination of sequences: eGFP, mSHROOM3 until T1387, and iLID + C450V mutation (C1739V in this construct). Highlighted.
