# Supplementary material for: Optogenetic control of apical constriction induces synthetic morphogenesis in mammalian tissues
Source: Nat Commun. 2022 Sep 14;13:5400. doi: 10.1038/s41467-022-33115-0 (PMC9474505; doi:10.1038/s41467-022-33115-0)
Supplement: Supplementary file 2 — Reporting Summary [file 41467_2022_33115_MOESM2_ESM.pdf]

nature research

Corresponding author(s): Miki Ebisuya

Last updated by author(s): Aug 29, 2022

Reporting Summary

Nature Research wishes to improve the reproducibility of the work that we publish. This form provides structure for consistency and transparency in reporting. For further information on Nature Research policies, see our Editorial Policies and the Editorial Policy Checklist.

Statistics

For all statistical analyses, confirm that the following items are present in the figure legend, table legend, main text, or Methods section.

n/a	Confirmed	
☐	☒	The exact sample size (*n*) for each experimental group/condition, given as a discrete number and unit of measurement
☐	☒	A statement on whether measurements were taken from distinct samples or whether the same sample was measured repeatedly
☐	☒	The statistical test(s) used AND whether they are one- or two-sided<br>*Only common tests should be described solely by name; describe more complex techniques in the Methods section.*
☒	☐	A description of all covariates tested
☒	☐	A description of any assumptions or corrections, such as tests of normality and adjustment for multiple comparisons
☐	☒	A full description of the statistical parameters including central tendency (e.g. means) or other basic estimates (e.g. regression coefficient) AND variation (e.g. standard deviation) or associated estimates of uncertainty (e.g. confidence intervals)
☐	☒	For null hypothesis testing, the test statistic (e.g. *F*, *t*, *r*) with confidence intervals, effect sizes, degrees of freedom and *P* value noted<br>*Give P values as exact values whenever suitable.*
☒	☐	For Bayesian analysis, information on the choice of priors and Markov chain Monte Carlo settings
☒	☐	For hierarchical and complex designs, identification of the appropriate level for tests and full reporting of outcomes
☒	☐	Estimates of effect sizes (e.g. Cohen's *d*, Pearson's *r*), indicating how they were calculated

Our web collection on statistics for biologists contains articles on many of the points above.

Software and code

Policy information about availability of computer code

Data collection	Olympus FV3000 Fluoview RS (Confocal microscope software). Leica Stellaris LAS X (Confocal microscope software).
Data analysis	FIJI 2.3.9/1.53q, MorphoGraphX 1.0, Python 3.7, DataViewerV2. Custom codes have been uploaded to GitHub ([https://github.com/mebisuya/OptoShroom3], with identifier (Zenodo, 10.5281/zenodo.7031023, 2022).

For manuscripts utilizing custom algorithms or software that are central to the research but not yet described in published literature, software must be made available to editors and reviewers. We strongly encourage code deposition in a community repository (e.g. GitHub). See the Nature Research guidelines for submitting code & software for further information.

Data

Policy information about availability of data

All manuscripts must include a data availability statement. This statement should provide the following information, where applicable:
- Accession codes, unique identifiers, or web links for publicly available datasets
- A list of figures that have associated raw data
- A description of any restrictions on data availability

Source data are provided with this paper. Due to the large file size, raw image data can be obtained upon request. Please contact Miki Ebisuya or Guillermo Martínez-Ara to request data. A response will be provided in less than 2 weeks.

Field-specific reporting

Please select the one below that is the best fit for your research. If you are not sure, read the appropriate sections before making your selection.

[x] Life sciences   [ ] Behavioural & social sciences   [ ] Ecological, evolutionary & environmental sciences

For a reference copy of the document with all sections, see nature.com/documents/nr-reporting-summary-flat.pdf

Life sciences study design

All studies must disclose on these points even when the disclosure is negative.

Sample size	No statistical method was used to predetermine the sample size. All experiments were performed at least three times, except for supp. video 11 (two times). Sample sizes were chosen based on practicality and throughput, and are consistent with previously published similar works in the field.
Data exclusions	In tissue folding experiments, some MDCK colonies on matrigel presented a migratory behaviour. Because this migration made them move out of the stimulation and imaging area, we decided to exclude them.
Replication	All attempts of replication have been successful. The variability in the extent of deformation was reported in the graphs or the movies showing all samples. All the experiments were independently replicated at least 3 times, except for supp. video 11 (two times).
Randomization	The experiments were not randomized since data analysis was automatic or semiautomatic.
Blinding	The Investigators were not blinded to allocation during experiments and outcome assessment. Data analysis was automatized to avoid possible biases.

Reporting for specific materials, systems and methods

We require information from authors about some types of materials, experimental systems and methods used in many studies. Here, indicate whether each material, system or method listed is relevant to your study. If you are not sure if a list item applies to your research, read the appropriate section before selecting a response.

Materials & experimental systems

n/a	Involved in the study	
[ ]	[x]	Antibodies
[ ]	[x]	Eukaryotic cell lines
[x]	[ ]	Palaeontology and archaeology
[x]	[ ]	Animals and other organisms
[x]	[ ]	Human research participants
[x]	[ ]	Clinical data
[x]	[ ]	Dual use research of concern

Methods

n/a	Involved in the study	
[x]	[ ]	ChIP-seq
[x]	[ ]	Flow cytometry
[x]	[ ]	MRI-based neuroimaging

Antibodies

Antibodies used	rabbit Non-muscle Myosin Heavy Chain II-B Antibody (dilution 1:200, Biolegend, 909902), anti-α-tubulin mouse mAb (dilution 1:400, Sigma, clone DM1A), alexa fluor 647 goat anti-rabbit (dilution 1:200, Invitrogen), and alexa fluor 647 goat anti-mouse antibody (dilution 1:200, Invitrogen) and phalloidin-atto 655 (dilution 1:200, Sigma).
Validation	These antibodies have been validated by providers:<br>According to the distributor, anti-α-tubulin mouse mAb (dilution 1:400, Sigma, clone DM1A) was shown to be reactive for human, gerbil, rat, chicken, mouse and validated for use in Immunoblotting, Immunofluorescence for the detection of α-Tubulin.<br><br>Rabbit Non-muscle Myosin Heavy Chain II-B Antibody (dilution 1:200, Biolegend, 909902) has verified Reactivity Human, Mouse, Rat. It has been validated by the provider for westernblotting and immunocytochemistry. For immunofluorescence, the following references are provided:<br>1. Ma X, et al. 2007. Mol Biol Cell. 18:2305. (IF)<br>2. Golomb E, et al. 2004. J Biol Chem. 279:2800. (WB, IF)<br>3. Hirota Y, et al. 2010. Development. 137: 3037. (IF)

April 2020

Eukaryotic cell lines

Policy information about cell lines

Cell line source(s)	MDCK cell line (MDCKII) was a gift from M. Murata lab, which was previously obtained from Dr. Kai Simons lab. Mouse ES cell line (EB5, Rx-GFP) was previously published by authors M. Eiraku and N. Takata in Nature 472, 51–56 (2011) (doi.org/10.1038/nature09941). Human iPS cell line IMR90-4 was purchased from WiCell.
Authentication	None of the cell lines were authenticated.
Mycoplasma contamination	All cell lines were tested every month for mycoplasma contamination and tested negative.
Commonly misidentified lines (See ICLAC register)	No commonly misidentified cell lines were used.
