# Supplementary material for: Optogenetic control of apical constriction induces synthetic morphogenesis in mammalian tissues
Source: Nat Commun. 2022 Sep 14;13:5400. doi: 10.1038/s41467-022-33115-0 (PMC9474505; doi:10.1038/s41467-022-33115-0)
Supplement: Supplementary file 15 — Description of Additional Supplementary Files [file 41467_2022_33115_MOESM15_ESM.pdf]

Title: Supplementary movie 1.
Description: Translocation of SspB-mCherryCShroom3 and apical constriction induced by stimulation of a single cell in an OptoShroom3-MDCK monolayer. After 20 minutes, the microscope was stopped for less than 1 minute to set more stimulation cycles, which caused a transient decrease in translocation. White polygon marks the stimulation area.

Title: Supplementary movie 2.
Description: Repeated stimulation of a single cell in an OptoShroom3-MDCK monolayer. iRFP-CAAX. Apical and basal slices. 3 stimulation rounds. White polygons mark the stimulation area.

Title: Supplementary movie 3.
Description: Stimulation of a group of cells in an OptoShroom3-MDCK monolayer on glass and 3D segmentation. iRFPCAAX. White rectangle marks the stimulation area.

Title: Supplementary movie 4.
Description: Stimulation of a group of cells in an OptoShroom3-MDCK monolayer on collagen. iRFP-CAAX. White rectangle marks the stimulation area.

Title: Supplementary movie 5.
Description: Simultaneous stimulation of two areas in an OptoShroom3-MDCK monolayer. iRFP-CAAX. White rectangles mark the stimulation area.

Title: Supplementary movie 6.
Description: Stimulation-induced folding of OptoShroom3-MDCK colonies and 3D rendering. GFP-NShroom3- iLID. Scale bar = 100 µm.

Title: Supplementary movie 7.
Description: Stimulation-induced folding of OptoShroom3-MDCK colonies with infra-red fluorescent beads labeling matrigel. GFP-NShroom3-iLID (green), fluorescent micro-beads (white).

Title: Supplementary movie 8.
Description: Retraction and coiling of cell sheets induced by selective stimulation of elongated OptoShroom3-MDCK colonies. Showing 5 different samples. GFPNShroom3-iLID. White rectangles mark the stimulation areas.

Title: Supplementary movie 9.
Description: Thickening of a neuroepithelium induced by selective stimulation of an OptoShroom3-optic vesicle organoid. GFP-NShroom3-iLID and Sspb-mCherry-CShroom3. White rectangles mark the stimulation area.

Title: Supplementary movie 10.
Description: Apical lumen reduction induced by selective stimulation of OptoShroom3-optic vesicle organoids. GFP-NShroom3- iLID signal. White polygons mark the stimulation areas.

Title: Supplementary movie 11.
Description: Microtubule organization during the thickening of a neuroepithelium induced by selective stimulation of an OptoShroom3-optic vesicle organoid. SiR-Tubulin. White polygon marks the stimulation area.

Title: Supplementary movie 12.
Description: Flattening of outer membranes induced by selective stimulation of OptoShroom3-neuroectodermal organoids, including live average quantification. Sspb-mCherryCShroom3. White polygons mark the stimulation areas.
